# Oral health status of community dwelling adults aged 50 years and over in Ireland. A cross-sectional analysis of the Wave 3 TILDA cohort.

**DOI:** 10.12688/hrbopenres.12891.1

**Published:** 2018-12-14

**Authors:** Amara Naseer, Jacinta McLoughlin, Orna A. Donoghue, Rose Anne Kenny, Brian O'Connell

**Affiliations:** 1 Dublin Dental University Hospital, Trinity College Dublin, Dublin, D02F859, Ireland; 2 The Irish Longitudinal Study on Ageing (TILDA), Trinity College Dublin, Dublin, D02PN40, Ireland; 3 Mercer's Institute for Successful Ageing, St James's Hospital, Dublin, Ireland

**Keywords:** ageing demographics, cohort studies, oral health, health surveys, public health dentistry

## Abstract

**Background**: Little is known about the current oral health status of adults in Ireland. The aim of this study was to measure the oral health status of community dwelling adults aged 50 years and over in Ireland, and to compare the current status to previous surveys of oral health in adults.

**Methods**: The Irish Longitudinal Study on Ageing (TILDA) Wave 3 provided an opportunity to assess the oral health of a subset of TILDA participants. Respondents attending for health assessments at the TILDA centre were offered an oral health examination. The World Health Organization examination criteria were used.

**Results: **Out of the 3111 people who were offered the oral health assessment (OHA), 2525 were examined. Adults below 50 years of age (n=17) and 4 respondents whose oral health data were unavailable at time of analysis were omitted, giving a final sample of 2504 respondents.  Among the OHA sample, 9.9% (249) were edentate; 11.5% (159) of females and 8% (90) of males. Of those aged 65 years and older, 15.6% were edentate compared with 40.9% in 2000-02. The mean number of teeth present in those aged 65 years or older was 14.9 for males and 14.2 for females compared with 9.9 and 7.4, respectively, in 2000-02. 56.8% of the dentate sample had 10 or more tooth contacts. The mean DMFT of those aged 50 years or more was 18.5 and the Root Caries Index (RCI) was 6.3. For adults aged 65 years and over, the mean DMFT decreased from 25.9 to 20.1 and the Root Caries Index decreased from 11.6 to 9.1, between 2000-02 and 2014-15.

**Conclusion**: The results suggest an improvement in oral health status of community dwelling adults aged 50 years and over in Ireland as compared to the previous Irish survey of 2000-02.

## Introduction

The ageing population is one of the great challenges that will confront health services in developed countries in coming years. It is estimated that by 2050, the number of adults aged 60 years and older worldwide will increase from 901 million to 2.1 billion, and the adults called “oldest old” (80 years and over) will more than triple (125 to 434 million), as compared to 2015 ^1^. In 2011, in Ireland, adults aged 65 years and over comprised 11.4% of the total population and this proportion is predicted to reach 22.4% in 2041. The proportion of adults aged 80 years or older is predicted to be 7.5% of the total population in 2046 ^2, 3^.

The ageing population faces the challenge of different chronic diseases including physical, mental and oral health related conditions. Oral health directly and indirectly mirrors the health of the entire body and mind, and poor oral health has been called a silent epidemic ^4^. It is impossible to be healthy without a healthy mouth because oral health has a bidirectional relationship with systemic physical and mental health ^5– 8^. For example, older adults may, as a result of poor oral function, eat a poor quality diet and avoid social interaction and in this way poor oral health may adversely affect health and wellbeing. Similarly, loss of physical and cognitive function, and increasing frailty, often result in less attention to oral health and reduced access to the care that is needed to maintain oral function.

Dental caries and periodontal disease are the most common chronic oral diseases affecting the adult population in Ireland and worldwide ^9, 10^, and the loss of natural teeth is considered to be a key indicator of poor oral health in older people ^11^. In older adults, understanding the value of oral health conditions such as the presence of natural teeth and their minimum functioning number, periodontal health, dental caries and tooth wear, is very important. The increased retention of natural teeth, and reductions in the levels of total tooth loss either due to dental caries or periodontal disease, are vital for the health and wellbeing of older adults. Although the complete loss of teeth with age has reduced dramatically in recent years, most oral health surveys have reported an age-related deterioration in oral health including an increase in tooth loss, poor periodontal health and increased tooth wear ^10, 12, 13^.

In Ireland, the last national survey on the oral health status of adults was undertaken in 2000–02, so up-to-date information reflecting the current oral health status is lacking. This makes it difficult to assess the needs of the population and to design services that maintain the oral health of older adults. The Irish Longitudinal Study on Ageing (TILDA) provided an opportunity to include an oral health assessment in community dwelling adults aged 50 years and over ^14^. The aim of this assessment was to provide an up-to-date picture of the oral health of older adults, and to compare the current data with previous data from Irish and international studies.

This oral health assessment (OHA) has not only provided an overview of current oral health status, it can form the basis for understanding oral health in the broader context of the health and social environment of older adults in Ireland. It may also serve as a baseline measurement for the longitudinal study of oral health, thereby increasing our understanding of the factors that specifically affect changes in the oral health of people in Ireland as they age.

## Methods

### Ethical considerations and consent

Ethical approval for this study was obtained from the Trinity College Faculty of Health Sciences Research Ethics Committee and participants provided written informed consent before the health assessment.

### Study design

TILDA is a large-scale, nationally representative, comprehensive cohort study on ageing in Ireland. It was started in 2009 and at the time of writing has completed its fourth wave of data collection ^15^. The TILDA cohort consists of randomly selected community-dwelling adults aged 50 years and over, although partners or spouses of any age can also participate. There are three modes of data collection, a computer aided personal interview (CAPI), self-completion questionnaire (SCQ) and health assessments ^14^.

In Wave 3 (March 2014 – December 2015), an optional oral health assessment (OHA) was included as part of the health assessment conducted in the TILDA health centre in Trinity College Dublin. As a dentist was not available during the full health centre opening hours, only those participants who completed a health assessment during the dentist hours were invited to participate in the OHA; there were no other exclusion criteria. Periodontal probing was omitted from the assessment of respondents at risk from bacteraemia. The examination criteria used in this study were the same as those used in previous Irish oral health surveys and similar to those recommended by WHO ^10, 16^. The examiners were trained by an experienced examiner (‘gold standard’--JMcL) from previous studies. A total of one trainer (‘gold standard’) and four assessors (including AN, BOC) completed the data collection. As the OHA was at the end of the health centre assessment (approximately three hours long), a maximum of 10 minutes was allocated for each OHA. Because of the time constraint, it was not possible to perform duplicate examinations during the main data collection. During the pilot phase, a calibration exercise was performed on non-participant volunteers, followed by dual assessment of study participants until any discrepancies between the trainer and assessors were resolved. For the oral health examination, standardised dental equipment consisted of a dental chair with floor mounted Daray LED examination light (Model- XL200 LED examination light, 12–30v/5.8–8.2w), standard plane dental mirror and WHO recommended Community Periodontal Index of Treatment Need (CPITN) probe-E ^16^. Methods and equipment were the same for all examinations. Standard cross infection control measures were followed during examinations.

All respondents attending for a health assessment were invited to participate in the OHA while an examining dentist was present. The data collected for the OHA was; number of natural teeth, use of dentures, CPITN, coronal caries at cavitation level and visual level (WHO and British Association for the Study of Community Dentistry-BASCD) ^17^, Root Caries Index of Katz ^18^, coronal tooth wear into dentine ^19^ and tooth contacts in the maximum intercuspal position (MIP) ^20, 21^. All criteria were based on visual examination and tactile sensing methods using a CPITN probe and no radiographs were taken. Data from the assessment was written on a paper form and then entered on a laptop computer and uploaded to the TILDA database.

For the purpose of the CPITN examination, the mouth was divided into sextants and the highest (worst) score in each sextant was recorded as the sextant score. The scores were; no disease (H), bleeding on examination (B), supra or sub gingival calculus present (C), pocket depth up to 4-5mm (P1), pocket depth >6mm (P2) and if no teeth were present in a sextant/unable to record (X) ^16^.

Lower tooth contacts in the MIP were recorded to evaluate the functionality of the dentition and the need for replacement of teeth ^21– 25^. To achieve MIP, participants were asked to swallow and keep their teeth closed together—the number of mandibular occlusal units in contact was counted. An occlusal unit is considered to be a single anterior tooth or premolar, or half a molar tooth (mesial or distal) ^25^. The percentages of dentate adults with fewer than 10 contacts, and 10 contacts or more, by age group and gender were calculated. Ten tooth contacts indicates approximately 20 teeth in occlusion, which is considered to be a minimal functioning dentition ^25^.

The prevalence of root caries was calculated using the Root Caries Index (RCI) among the dentate adults ^18^. This index gives the proportion of exposed roots with caries or restorations due to caries (RCI = mean decayed and filled roots /mean exposed roots %). Decayed and filled roots were recorded at tooth level rather than surface level, and so RCI was also calculated at tooth level.

Tooth wear was recorded by visual examination. The Bardsley tooth wear index was used in this study to record coronal tooth wear into dentine ^19^. The mouth was divided in to sextants and each sextant was individually scored. Tooth wear was recorded as; no wear, exposed dentine comprised <1/3 of worst surface of a tooth, exposed dentine comprised >1/3 of worst surface of a tooth, or the sextant was excluded, as no teeth were present in the sextant or unable to record a score. The worst tooth in a sextant was recorded as a sextant score. The highest score of dentine wear per person was recorded as a person’s tooth wear level.

During the statistical analysis, some oral health indicators were calculated for the full OHA sample (denture wear, number of teeth and mean DMFT (decay-missing-filled teeth)) and other oral health indicators (tooth contacts, RCI and periodontal health) were calculated for the dentate sample only, resulting in two bases for results. “Base edentate/dentate” means statistical analysis involved the full OHA sample, including edentate and dentate adults, whereas “Base dentate” means statistical analysis was run only on the dentate sample.

TILDA is subject to the legislation under the Data Protection Act 1988 and the Data Protection (Amendment) Act 2003. All data protection protocols were followed during collection, processing, analysis and reporting on data ^26^. Data analysis followed completion of data cleaning by accessing a TILDA hot desk at the TILDA research centre in Trinity College. STATA software (Stata 14.1 Stata Corp LLC Texas USA) was used for data analysis.

## Results

### Oral health assessment sample selection

The OHA sample is a sub-sample of the respondents who attended a TILDA health centre assessment at Wave 3. A total of 4309 respondents attended for the health assessment, of whom an opportunistic sample of 3111 (72.2%) were invited to have the OHA, and of these 2525 (81.1%) agreed to the assessment. Those aged less than 50 years (n=17) were omitted from this analysis. The full OHA sample consisted of 2508 respondents, however, as the data for 4 of these respondents was not available at the time of the analysis, the results reported here are for 2504 respondents ^15^.

### Oral health assessment sample vs TILDA sample (population sample)

The TILDA cohort is a nationally representative sample of adults aged 50 years and over (a population sample). To reduce the bias of a non-random response, weights can be applied to ensure that a sample is representative of the target population, however, as the OHA was completed by a sub-sample of the cohort, it was not possible to apply weights to the OHA sample. Table 1 below reports the two sample proportion test (Z test) for comparisons of the characteristics between the full OHA sample and the Wave 3 TILDA cohort. For the purpose of comparison between the different samples, 10 variables with 23 categories were selected. There was no difference between the samples (OHA and TILDA) in 8 out of the 23 categories.

**Table 1.** Comparison of the oral health assessment sample with the TILDA sample by a two sample proportion test (Z test). P values less than 0.05 are shown in bold.

Characteristic	Oral Health Assessment Sample (n=2508) n (%)	Population (TILDA sample) (n=6618) n (%)	Hypothesis test of proportions Population (TILDA) vs OHA
Age group			**P value**
50–64 years	1219 (48.6)	3036 (45.9)	**0.0196**
65–74 years	918 (36.6)	2110 (31.9)	**< 0.001**
≥75 years	371 (14.8)	1472 (22.2)	**< 0.001**
Female	1386 (55.3)	3679 (55.6)	0.7786
Education level			
Primary	478 (19.1)	1737 (26.3)	**< 0.001**
Secondary	1005 (40.1)	2610 (39.4)	0.5805
Tertiary/higher	1024 (40.8)	2269 (34.3)	**< 0.001**
Marital status			
Married	1889 (75.3)	4573 (69.1)	**< 0.001**
Never married	168 (6.7)	562 (8.5)	**0.0048**
Separated/ divorced	192 (7.7)	469 (7.1)	0.3494
Widowed	259 (10.3)	1014 (15.3)	**< 0.001**
Locality			
Dublin	681 (27.1)	1592 (24.1)	**0.0023**
Other urban	661 (26.4)	1840 (27.8)	0.1664
Rural	1166 (46.5)	3186 (48.1)	0.1588
Grew up in rural area	1440 (57.4)	3891 (58.8)	0.2331
Never lived abroad	669 (26.7)	1534 (23.2)	**0.0005**
Current or former smoker	1302 (51.9)	3609 (54.5)	**0.0251**
No health insurance or medical card	209 (8.3)	591 (8.9)	0.3681
Self-reported health			
Excellent	394 (15.7)	921 (14.2)	**0.0294**
Very good	897 (35.8)	2169 (33.4)	**0.0069**
Good	851 (34.0)	2227 (34.2)	0.8001
Fair	312 (12.4)	970 (14.9)	**0.0065**
Poor	52 (2.1)	215 (3.3)	**0.0029**

In summary, the OHA sample was younger, more respondents were married, they were better educated, with good to excellent self-rated general health and more likely to be living in Dublin, as compared with the TILDA (population) sample. As the OHA sample size is large and there are small differences in baseline characteristics between the OHA sample and TILDA cohort, the oral health findings should be representative of the overall sample.

### Oral health assessment sample description

For the analysis, the OHA sample was stratified into three age groups as recommended by WHO ^16^; 50–64 years old, 65–74 years old and 75 years and over (see Table 2 for gender breakdown). Almost half of the sample was aged 50–64 years with 14.8% aged 75 years and over. Overall, the OHA sample consisted of more females than males (55.3% vs 44.7%), but this gender difference decreased with increasing age.

**Table 2.** The oral health assessment sample by gender and age group (n=2504).

Gender	Age groups			Total
	50–64 years	65–74 years	75 years and over	
**Male**	511	422	187	1120
	42.0%	46.1%	50.5%	44.7%
**Female**	707	494	183	1384
	58.0%	53.9%	49.5%	55.3%
**Total Row %**	1218	916	370	2504
	48.6%	36.6%	14.8%	100%

### Dentate/edentate proportion

Adults with at least one natural tooth present were recorded as dentate. Table 3 shows that overall, 9.9% of the sample were edentate (no teeth).

**Table 3.** Number and percentage of edentate/dentate sample by age group and gender (Base edentate/dentate, n=2504).

Age group	50–64 years		65–74 years		75 years & over		Total
Gender	Male	Female	Male	Female	Male	Female	
**Edentate**	15	33	36	71	39	55	249
	2.9%	4.7%	8.5%	14.4%	20.9%	30.1%	9.9%
**Dentate**	496	674	386	423	148	128	2255
	97.1%	95.3%	91.5%	85.6%	79.1%	69.9%	90.1%

The proportion of edentate adults increased with age, and more females were edentate than males in all age groups. Table 3 also shows the increase in edentulism with age was greater in females than males. Among the sample, the proportion of adults who were completely edentate, only edentate in the upper arch, and only edentate in the lower arch was also calculated ( Table 4).

**Table 4.** Number and percentage of adults edentate in both arches, edentate in the upper arch, edentate in the lower arch, and partially dentate in both arches (Base edentate/dentate, n=2504).

	Upper & lower arch edentate	Upper arch edentate	Lower arch edentate	Dentate or partially dentate in both arches
**n**	249	350	477	1428
**%**	9.9%	14.0%	19.0%	57.0%

57% per cent of adults had one or more teeth in both arches (dentate or partially dentate), and a higher proportion of the sample was edentate in the lower arch, only compared with those edentate in the upper arch only and edentate in both arches.

### Denture wear

Table 5 shows that 46.9% of the sample had some type of removable denture, 18.4% were wearing a complete upper denture (with or without another denture) while the remaining 28.4% had other combinations of partial dentures and complete dentures. While 9.9% of the sample was edentate, only 9.1% were wearing complete dentures for the upper and lower arches.

**Table 5.** The proportion of denture wearers by age group and type of denture (Base edentate/dentate, n=2504).

Age group	No upper or lower denture		Complete upper and lower dentures		Complete upper and partial lower dentures		Complete upper denture only		All other combinations of complete and partial dentures		Total
	n	%	n	%	n	%	n	%	n	%	n
**50–64** **years**	838	68.8	42	3.5	10	0.8	41	3.4	287	23.6	1,218
**65–74** **years**	392	42.8	98	10.7	51	5.6	67	7.3	308	33.6	916
**75 years** **and over**	100	27.0	88	23.8	24	6.5	41	11.1	117	31.6	370
**Total**	1,330	53.1	228	9.1	85	3.4	149	5.9	712	28.4	2504

The percentage of adults wearing dentures increased with age irrespective of the type of denture. With increasing age, more adults had also lost their remaining teeth, and moved from partial to complete dentures. For example, in age group 50–64 years, 31.2% were denture wearers whereas in those aged 75 years and over, 73% were denture wearers and 23.8% had complete dentures.

### Number of teeth

**Figure 1.**
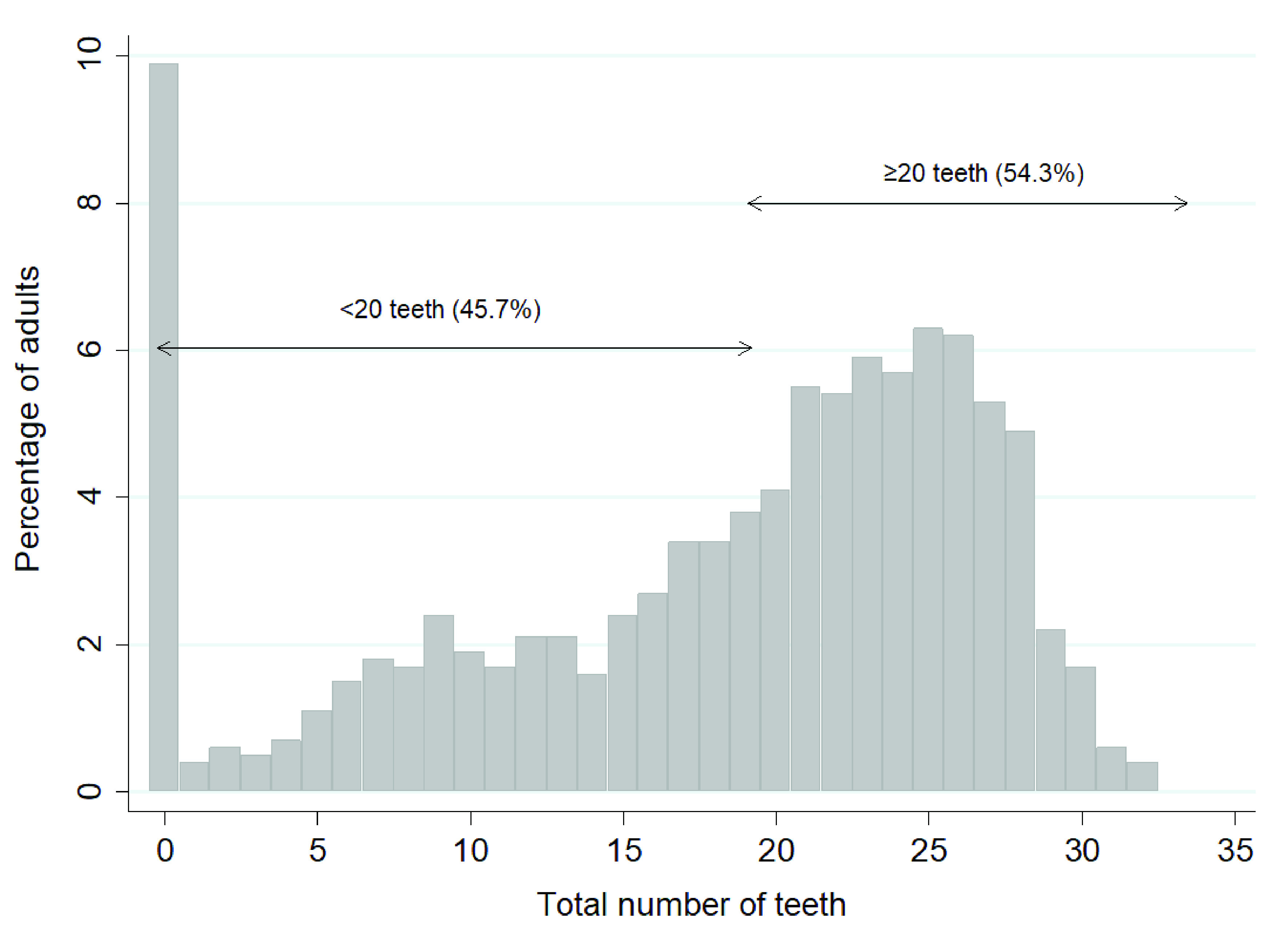
Percentage of adults by total number of teeth and the proportion with <20 teeth and ≥ 20 teeth. (Base edentate/dentate, n=2504). Mean number of teeth was 17.9, SD = 8.9, Median=21.

Figure 1 shows the frequency distribution of the number of teeth present in the OHA sample. It shows that 9.9% of adults had no teeth, and 54.3% of adults had 20 or more teeth. When the results in Table 6 are compared, it can be observed that although a higher proportion of females were edentate, females in the youngest age group, who were dentate, had a slightly higher mean number of teeth than males (21.5 vs 21.1). This suggests that the trend in higher tooth loss in older women in Ireland may be reversing, although this would need to be substantiated by a longitudinal study.

**Table 6.** Mean number of teeth per person by age group and gender (Base edentate/dentate, n=2504).

Age groups	Male	Female	Total
**50–64 years**	21.1	21.5	21.3
**65–74 years**	16.3	15.4	15.8
**75 years & over**	11.9	11.1	11.5
**Total**	17.7	17.9	17.9

### Tooth contacts

**Figure 2.**
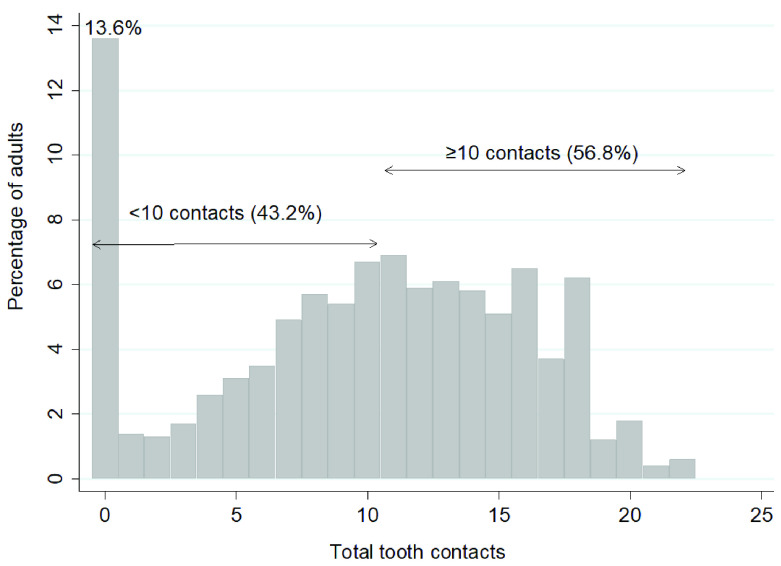
Percentage of adults by total number of tooth contacts and with <10 tooth contacts and ≥10 contacts (Base dentate, n=2255). Mean 9.9, SD= 5.9, Median= 11.

The percentage of dentate adults with fewer than 10 occlusal contacts, and 10 contacts or more, by age group and gender was calculated. Figure 2 shows that 56.8% of the dentate sample had 10 or more tooth contacts. Notably, 13.6% of dentate adults had no contacts; these adults were edentate in one arch, wearing dentures, had cross bites, teeth not in contact with other teeth, or just roots remaining. Table 7 shows that the proportion of dentate adults with 10 or more contacts decreased with age and was higher in females, although the gender difference narrowed in the older age groups.

**Table 7.** Number and percentage of adults with fewer than 10 tooth contacts, and equal to or more than 10 tooth contacts, by age group and gender (Base dentate, n=2255).

Age group	50–64 years		65–74 years		75 years & over		Total
Gender	M	F	M	F	M	F	
**<10 Contacts**	172	179	214	225	101	84	975
	34.7%	26.6%	55.4%	53.2%	68.2%	65.6%	43.25%
**≥ 10 Contacts**	324	495	172	198	47	44	1280
	65.3%	73.4%	44.6%	46.8%	31.8%	34.4%	56.75%

### Decayed, missing and filled teeth (DMFT)

Dental caries was recorded at cavitation (DMFT-c) and at visual caries level (DMFT-v). As the results indicated a difference of 0.1 between DMFT-c and DMFT-v (DMFT-c= 18.5, DMFT-v =18.6), it was decided to only report DMFT-c.

During the OHA it was possible to identify that some teeth were missing for reasons other than dental caries. These were missing premolars with no residual spaces (orthodontic extractions or congenital absence), third molars extracted due to impaction and teeth lost due to trauma. Where there was certainty about the reasons for loss these teeth they were recorded as missing for other reasons and not counted in the M component of DMFT. Where it was unclear, or there was doubt about the reasons for tooth loss, these teeth were recorded as missing due to caries. Similarly, in the edentate group third molar teeth were not recorded as missing due to caries. For this reason, the maximum DMFT score for edentate adults is shown as 28 but for dentate adults is 32.

**Figure 3.**
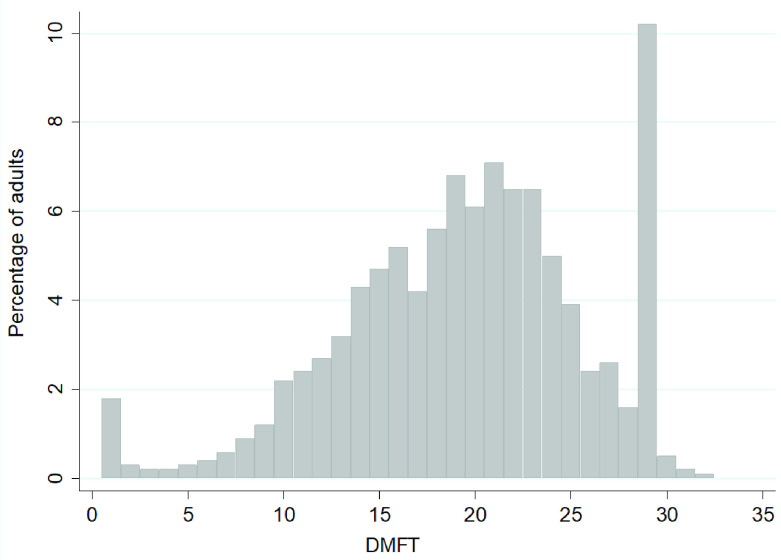
Frequency distribution of the percentage of adults aged 50 years and over by DMFT score (Base edentate/dentate, n=2504). Mean= 18.5, SD= 6.3, Median=19.

**Figure 4.**
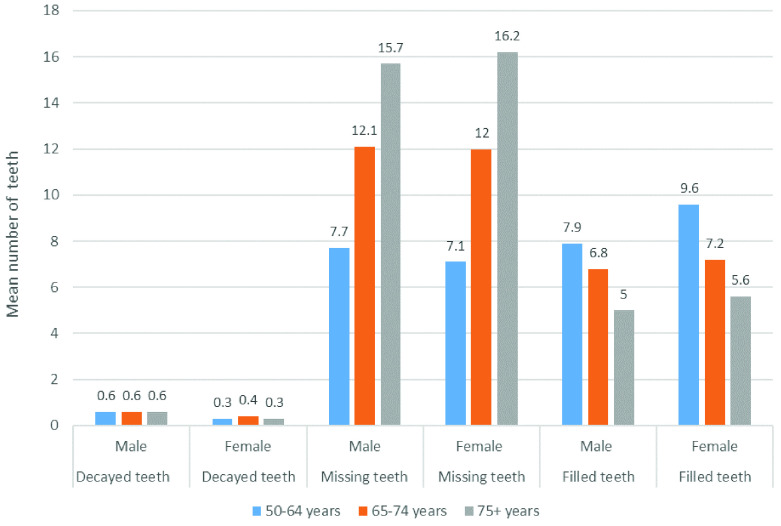
Mean decayed, missing and filled teeth components of DMFT by age group and gender (Base edentate/dentate, n=2504).

Figure 3 shows that overall, 10.22% of adults had a DMFT score of 28, which includes the 9.9% who were edentate. The DMFT scores are negatively skewed with a greater percentage of adults having high values for DMFT. Only 1.8% of adults had a DMFT score of 1. Mean DMFT values by age group and gender are reported in Table 8. There was a difference of 4.9 in the mean DMFT between age group 50–64 years, and age group 75 years and over. Overall, females had a slightly higher mean DMFT (0.3) score than males, and this gender difference was present among all three age groups. Figure 4 shows the contribution of the decayed teeth, missing teeth, and filled teeth components of DMFT by age group and gender.

**Table 8.** Mean DMFT level by age group and gender (Base edentate/dentate, n=2504).

Age groups	Male	Female	Total DMFT
**50–64 years**	16.4	17.0	16.7
**65–74 years**	19.4	19.6	19.5
**75 years & over**	21.3	22.0	21.6
**Total**	18.3	18.6	18.5

Of the total mean DMFT of 18.5, the contribution of decayed teeth was 0.6 in males and 0.3 in females, and was almost the same in all age groups. When compared to men, women in all age groups had fewer decayed teeth, more filled teeth, and women aged 75 years and over had more missing teeth, which suggests that women access treatment more. It is notable that in the youngest age group, the proportion of missing teeth was much lower than in the two older age groups, with corresponding higher proportions of filled teeth and the same proportion of decayed teeth. It remains to be seen whether this younger cohort can maintain more of their natural teeth as they age, as this would represent a major shift in the oral health of older adults in Ireland.

### Root caries

Root caries is reported as the mean number of decayed and filled roots as a proportion of the mean number of exposed roots with recession ( Table 9). There was an increase in RCI (4.3 vs 10.2) and the mean number of decayed/filled roots (0.5 vs 1.1) and a decrease in the mean number of exposed roots (11.1 vs 10.3) with increasing age. This decrease in exposed roots may be due to the lower number of teeth with increasing age. Females aged less than 75 years had higher levels of root caries than males, but those aged 75 years and over had slightly lower levels than males.

**Table 9.** Mean number of exposed roots, mean decayed/filled roots and Root Caries Index (RCI) by age group and gender (Base dentate, n=2255).

Age groups	Mean decayed/filled roots (DFR)			Mean exposed roots (ER)			RCI		Total RCI
	Male	Female	Total	Male	Female	Total	Male	Female	
**50–64 years**	0.4	0.5	0.5	11.6	10.7	11.1	3.6	4.9	4.3
**65–74 years**	0.8	0.9	0.9	11.1	10.7	10.8	7.2	8.3	7.8
**75 years & over**	1.1	1.0	1.1	10.1	10.6	10.3	10.6	9.8	10.2

### Periodontal health

The CPITN was used for the periodontal health assessment. The severity of periodontal disease was reported by the maximum CPITN score per person, and the extent as the mean number of sextants affected by the different scores for the dentate adults. Table 10 shows that the proportion of men and women with completely healthy periodontal tissues or bleeding gingivae or deep pockets was low, at less than 5%, in all age groups. The majority of older adults needed simple treatment for calculus and shallow pockets. The gender differences were small, with females tending to have better periodontal health status.

**Table 10.** Percentage and total number of adults with a maximum CPITN score of H (healthy), B (bleeding), C (calculus), P1 (shallow pocket), P2 (Deep pocket) and X (missing sextant) by age groups and gender (Base-Dentate n=2255).

Age groups	H%		B%		C%		P1%		P2%		X%		Total n
	M	F	M	F	M	F	M	F	M	F	M	F	
**50–64 years**	1.4	3.9	1.1	2.0	10.9	18.4	24.3	30.6	4.1	2.5	0.6	0.3	1170
**65–74 years**	2.4	3.5	0.9	2.7	14.3	19.3	24.9	23.9	2.7	2.2	2.6	0.7	809
**75 years and over**	3.6	4.7	3.3	1.8	15.9	19.9	22.1	17.4	2.5	1.5	6.2	1.1	276
**Total n**	45	86	29	50	288	426	546	599	77	51	45	13	2255

Table 11 shows the mean number of sextants per person affected by the different CPITN scores, stratified by age group and gender. In Table 10, it could be seen that less than 5% of the sample had deep periodontal pockets, while Table 11 shows that the mean number of sextants with deep pockets is also small (0.1–0.2). With respect to shallow periodontal pockets, the mean number of sextants affected was between 0.8 and 1.5, which suggests that pockets were not very extensive in this sample. The results from these two tables indicate that the periodontal treatment needs of this sample were neither complex nor extensive.

**Table 11.** Mean number of sextants per person affected by different CPITN score: H (healthy), B (bleeding), C (calculus), P1 (shallow pocket), P2 (Deep pocket) and X (missing sextant) among dentate sample by age group and gender (Base dentate, n=2255).

Age groups	H		B		C		P1		P2		X	
	M	F	M	F	M	F	M	F	M	F	M	F
**50–64 years**	1.6	2.2	0.6	0.7	1.4	1.3	1.5	1.2	0.2	0.1	0.7	0.5
**65–74 years**	1.4	1.9	0.5	0.5	1.4	1.3	1.1	0.8	0.1	0.1	1.5	1.4
**75 years & over**	1.4	1.7	0.4	0.4	1.1	1.2	0.9	0.8	0.1	0.1	2.2	1.8

### Tooth wear

Table 12 shows that in all age groups, fewer than 7% of respondents had no wear into dentine, while 50.6% had wear into dentine of less than one third of the worst surface. In all three age groups, there was more severe wear in males compared with females. In the age groups included in this study, some tooth wear would be considered to be physiological and for this reason, the low percentage with no wear is not unexpected.

**Table 12.** Percentage of adults with no tooth wear, dentine exposed less than 1/3 of worst surface, dentine exposed more than 1/3 of worst surface, or sextant excluded, among the dentate sample by age group and gender (Base dentate, n=2255).

Age groups	No wear		Wear <1/3 of dentine		Wear >1/3 of dentine		Excluded		Total
Gender	M	F	M	F	M	F	M	F	n
**50–64 years**	3.3	6.9	20.3	32.3	18.5	17.7	0.3	0.7	1170
**65–74 years**	3.3	5.4	20.2	28.1	23.9	18.4	0.5	0.4	809
**75 years and** **over**	2.5	4.7	23.9	25.4	26.1	16.3	1.1	0	276
**Total n**	73	138	465	675	482	401	10	11	2255

### National and international comparisons

In Ireland, there have been considerable improvements in the oral health status of adults as compared to previous Irish oral health surveys conducted in 1989–1990 ^27^ and 2000–2002 ^10^. The age group 65 years and over was characterised in each of the surveys. A summary of the principal oral health indicators in older adults in Ireland, from the last three surveys, is shown in Table 13. Caution is required when comparing the mean DMFT values as in this age group the two previous surveys used the WHO methodology, including teeth missing for all reasons in the calculation of mean DMFT. As explained above, in this study it was possible in some situations to identify teeth lost for reasons other than dental caries.

***Edentulism.*** Table 13 indicates that in Ireland, among adults aged 65 years and over, the prevalence of edentulism has decreased by more than two-thirds from 1989–90 to 2014–15 and most of that decrease has occurred since 2000–02. Though the prevalence of edentulism has decreased more dramatically in females compared to males, in 2014–5 the prevalence was still about 50% higher in females. A comparison of Irish and international prevalence of edentulism is shown in Table 14.

**Table 13.** Changes in edentulism, mean number of teeth, DMFT and RCI among adults aged 65 years and over in Ireland from 1989–90 ^27^ to 2000–02 ^10^ and in the current study, 2014–15.

Oral health indicators	Examination Year	Male	Female	Total
**% Edentate**	**2014–15**	12.3	18.6	15.6
	**2000–02**	34.6	45.6	40.9
	**1989–90**	33	61	48
**Mean number of teeth**	**2014–15**	14.9	14.2	14.6
	**2000–02**	9.9	7.4	8.5
	**1989–90**	10.1	4.9	7.3
**Mean DMFT**	**2014–15**	20.0	20.2	20.1
	**2000–02**	24.8	27	25.9
	**1989–90**	25.6	28.8	27.3
**RCI**	**2014–15**	9.1	9.1	9.1
	**2000–02**	12.7	10.6	11.6
	**1989–90**	20.9	14.9	18.5

**Table 14.** Percentage edentulism by age groups, country and examination year.

Country	Year of Examination	Age group	% Edentate
** Ireland**	**2014–15**	50–64 years 65–74 years 75 years and over	3.9 11.7 25.4
**UK ^28^**	**2009**	55–64 years 65–74 years 75–84 years 85 years and over	5 15 30 47
**USA ^29^**	**2012**	65–74 years 75 years and over	13 25.8
**Australia ^30^**	**2006**	55–74 years 75 years and over	13.9 35.7
**New Zealand ^31^**	**2009**	55–64 years 65–74 years 75 years and over	14.5 29.6 39.6

There are similar trends in the prevalence of edentulism with age in different countries, although the age groups and dates of the studies vary. For example, in the age group 75 years or older, the TILDA OHA sample in 2014–15 had a similar prevalence of edentulism when compared to the USA in 2012. For the group aged 65–74 years, edentulism in the TILDA OHA sample (11.7% in 2015) and in the UK (15.0% in 2009) are comparable taking account of the time difference between the studies.

***Mean number of teeth.*** Table 13 above shows that there has been a doubling in the mean number of teeth in adults aged 65 years and older from 1989–90 to 2014–15 (7.3 to 14.6) in Ireland. This trend in females is particularly positive where the mean number of teeth almost doubled between 2000-02 and 2014–15. Although mean number of teeth is a crude measure of oral health status, as it gives no indication of the condition of these teeth, it is nonetheless a positive trend that natural teeth have been retained rather than extracted.

Table 15 shows that adults in Ireland, aged 65–74 years and 75 years and over, had a lower mean number of teeth when compared to the UK, New Zealand and Australia, despite the fact that the studies in these countries were completed 5–10 years before the TILDA study. Together, these findings suggest that in Ireland, fewer adults above 65 years may be completely edentate compared to this group of countries, but the mean number of teeth per person is also less when compared to the UK, Australia and New Zealand. A possible explanation may be the different methods used in these studies. In this study the mean number of teeth was calculated for the whole sample (edentate and dentate) whereas the studies of UK, Australia and New Zealand calculated mean number of teeth among dentate sample only.

**Table 15.** Mean number of teeth per person by age groups, country and year of examination.

Country	Year of examination	Age group	Mean number of teeth
**Ireland**	**2014–15**	50–64 years 65–74 years 75 years and over	21.3 15.8 11.5
UK * ^28^	**2009**	55–64 years 65–74 years 75–84 years 85 years and over	23.2 20.9 17.1 14.0
**Canada (CHMS) ^32^**	**2007–09**	40–59 years 60–79 years	24.1 19.4
New Zealand * ^31^	**2009**	55–64 years 65–74 years 75 years and over	24.0 19.7 18.1
Australia * ^30^	**2004–06**	65–74 years 75 years and over	21.8 17.9

*Dentate sample only

***Dental caries.*** There was a decrease in mean DMFT from 1989–90 to 2014–15, for those aged 65 years and older in Ireland (27.3 vs 20.1), as shown in Table 13. It is notable that most of this decrease in DMFT occurred since 2000–02. From 1989-90 to 2014–15, the gender difference in mean DMFT also reduced from 3.2 to 0.2.

Although there were differences in the calculation of the M component of DMFT in this study compared to 1989–90, the proportion of untreated decay in the DMFT decreased from 4% to 2.4%, and the proportion of filled teeth increased from 6% to 32.2% in the DMFT (data not shown). If the mean DMFT values were calculated in the same way, then these differences would be even greater. These findings, along with the doubling in the mean number of teeth ( Table 13), suggest that adults over 65 years are not only keeping more of their teeth, but these teeth are in a healthier state. It also appears that these adults have accessed dental care which is more oriented to the maintenance of teeth compared to the past.

Table 13 also indicates that RCI reduced by more than half in Ireland from 1989–90 to 2014–15 (18.5 vs 9.1). The gender difference for RCI was reduced from 6 in 1989–90 to 0 in 2014–15. This is an encouraging finding, as it was thought that the prevalence of root caries in older people might increase with increased retention of natural teeth. This is because teeth tend to lose periodontal attachment with age and exposed root surfaces are vulnerable to the development of root caries.

Comparison of the mean DMFT among the TILDA OHA sample and other countries is shown in Table 16. It should be noted that in both the Australian and New Zealand surveys these mean DMFT values were for the dentate population as edentate people were excluded from the oral health assessments.

The TILDA OHA sample had a lower mean DMFT when compared to data from New Zealand and Australia, however, these studies took place 5–10 years before the Irish study which might account for some of this difference in mean DMFT. The inclusion of the edentate group in the TILDA calculation of mean DMFT would suggest that the difference in mean DMFT between Ireland and Australia and New Zealand may in fact be greater than it appears in this table.

**Table 16.** Mean DMFT by age groups, country and examination year.

Country	Year of Examination	Age group	Mean DMFT
** Ireland**	**2014–15**	50–64 years 65–74 years 75 years and over	16.7 19.5 21.6
New Zealand * ^31^	**2009**	55–64 years 65–74 years 75 years and over	21.7 24.2 24.8
Australia * ^30^	**2004–06**	55–64 years 65–74 years 75 years and over	21.7 23.2 24.6

*Dentate sample only

***Periodontal health.*** In Ireland, the changes over time in the proportion of adults aged 65 years and over with maximum CPITN score from 2000–02 to 2014–15 are shown in Table 17. From 2000–02 to 2014–15, the proportion of adults with a CPITN score of ‘healthy’ has slightly reduced. There has also been a noticeable increase in the proportion of people with calculus and shallow pockets, but a substantial reduction in the proportion with deep pockets. It is also important to note the fall in the number of excluded sextants (X), which indicates that more sextants had the minimum number of teeth for the CPITN examination to be carried out. The increase in the proportion with calculus and shallow pockets probably also reflects increased tooth retention.

**Table 17.** Changes over time in percentage of adults with maximum value of CPITN-severity score of H (healthy), B (bleeding), C (calculus), P1 (shallow pocket), P2 (deep pocket) and X (missing sextant) among dentate adults 65 years and over in Ireland (Base dentate).

Year of examination	H	B	C	P1	P2	X	Total (n)
**2014–15**	6.6	4.0	35.1	46.4	4.7	4.3	1085
**2000–02**	6.9	3.6	29.5	37.6	12.0	10.1	390

## Discussion

Data on the health of the population is a key part of identifying needs, planning public health strategies and assessing the effectiveness of public health policies. This is especially true of oral health, which is sensitive to socioeconomic conditions, dietary trends, lifestyle and access to care. At the same time, most dental disease is preventable so there is the potential for improvement in oral health, and by extension general health, at relatively low cost ^33^. The last national survey of adult oral health in Ireland was conducted in 2000–02 and the results were published in 2007. With a new national oral health policy imminent, it is critical to understand the current oral health status of older adults in Ireland. The TILDA study was a valuable opportunity to examine the oral health of a nationally representative cohort of older Irish adults: this cohort has been extensively characterised in terms of their physical and mental health, wellbeing, social interactions, and socioeconomic status.

The respondents who participated in the OHA were similar to the whole TILDA cohort in the key areas of gender, medical card status, urban-rural dwelling, and self-reported health status. Compared to the nationally representative TILDA cohort, the OHA group had more respondents from Dublin, were younger, and were more likely to have tertiary/higher level education. Comparing this study to previous surveys, the prevalence of edentulism has continued to decline rapidly in Irish adults, following the trend seen in many other English-speaking countries, though it was noted that older women are still more likely to have no teeth at all compared to men. The comparison also highlights economic, cultural, and historic differences between countries with respect to edentulism; for example New Zealand had a much higher prevalence of edentulism than the UK at the same time point, perhaps due to a health service that emphasised the provision of dentures ^31^. While decreasing as a proportion of the Irish population, edentate adults are at least 4 times more likely to have a difficulty with everyday functions (smiling, speaking, eating) compared to those with natural teeth ^34^. Edentate adults are also more likely to suffer from depressive symptoms and loneliness, while having a lower level of social participation ^34^. There was little difference between men and women who had teeth, with regard to the number, condition, and functional capacity.

Having at least 20 natural teeth, or 10 pairs of contacting teeth, is considered a benchmark of a functional dentition, that is a dentition that will generally provide adequate functional capacity and may not require additional prosthetic teeth ^25^. This study found that 56.8% of the OHA sample had 10 or more pairs of contacting teeth, though as with other variables, there was a marked difference between the younger and older age groups. This means that there is a much larger need for intervention in the form of replacement teeth among older adults in Ireland, though a report on this cohort found that people with fewer teeth were actually less likely to access dental services ^34^.

One of the concerns raised about maintaining oral health in older people is that, paradoxically, the retention of natural teeth needs more ongoing care than a complete loss of teeth. Without adequate regular plaque control, a healthy diet and some dental intervention, natural teeth can be susceptible to caries and periodontal disease which leads to pain, infection and loss of function. The susceptibility of older adults to dental disease may be heightened due to periodontal recession and an increase in medications that cause dry mouth. In Ireland, adults who become frail, dependent, or live in residential care will often lose access to dental services, except in an emergency. This study found a low level of untreated crown and root caries, and mostly mild periodontal disease, which suggest that independently-living older adults can maintain their oral health fairly well. Nonetheless, the level of root caries in those aged 75 years or older was double that of the younger age groups. The challenge will be to provide adequate care for the growing number of frail older people who, in the future, will have a far greater number of natural teeth.

This study made a broad assessment of oral health, using a methodology that allows for comparison to previous national oral health surveys and similar international studies. Other strengths of the study design were the large sample size and the extensive information collected during the CAPI, SCQ and health assessment. This will be extremely useful in further analysis that will link the objective measures of oral health with other health outcomes. The OHA sample also had similar characteristics to the TILDA cohort, suggesting that the findings should be representative of the overall sample. However, the study design had some limitations. The OHA participants were recruited from those attending a health centre assessment in Dublin; at Wave 1, participants who had a home-based assessment or did not complete a health assessment were older and had poorer health indicators than those who did attend a health centre ^35^, so it is likely that the oral health status of these participants is also poorer than the current OHA sample. Therefore, while it should be possible to generalise these results to the majority of the community-dwelling population aged 50 years and over, they may underestimate the prevalence and extent of oral health problems in adults with poorer health and those in residential care.

## Conclusions

Data from this study shows that there has been considerable improvement in the retention of teeth among community dwelling adults aged 50 years and over in Ireland, as compared to previous Irish surveys. Since 1989-90, the proportion of edentate adults aged 65 years and over has decreased by two-thirds, and the mean number of natural teeth has more than doubled. However, tooth loss was still common among older adults, posing a challenge for our health services. Currently, there is little service provision for dentate older adults, especially those who become frail or dependent, suggesting the need for a qualitative change in the oral care for older people in Ireland.

If extrapolated to the whole population, these findings suggest that Ireland has more slowly followed international trends for improvement in the oral health status of older adults, observed in other developed countries like the UK, USA, Australia and New Zealand. These trends are very positive but they indicate a requirement for more maintenance care, restorative and periodontal treatment, and less need for complete dentures than previously. The findings of this study should provide a valuable resource for oral health policy and planning of oral health services for older people in Ireland.

## Data availability

The data presented in this report was collected during Wave 3 of TILDA. Wave 3 data is available from the Irish Social Science Data Archive (ISSDA):

ISSDA: Dataset 1. The Irish Longitudinal Study on Ageing (TILDA) Wave 3, 2014–2015. Study number: 0053-04. www.ucd.ie/issda/data/tilda/wave3. ^36^

### Accessing the data

To access the data, please complete a ISSDA Data Request Form for Research Purposes, sign it, and send it to ISSDA by email ( issda@ucd.ie).

For teaching purposes, please complete the ISSDA Data Request Form for Teaching Purposes, and follow the procedures, as above. Teaching requests are approved on a once-off module/workshop basis. Subsequent occurrences of the module/workshop require a new teaching request form.

Data will be disseminated on receipt of a fully completed, signed form. Requests to access the OHA data should be made directly to TILDA ( tilda@tcd.ie)

## Consent

Ethical approval for this study was obtained from the Faculty of Health Sciences Research Ethics Committee in Trinity College Dublin and participants provided written informed consent before the health assessment.
