# Dental health status of community-dwelling adults aged 50 years and over in Ireland. A cross-sectional analysis of the Wave 3 TILDA cohort.

**DOI:** 10.12688/hrbopenres.12891.3

**Published:** 2022-02-08

**Authors:** Amara Naseer, Jacinta McLoughlin, Orna A. Donoghue, Rose Anne Kenny, Brian O'Connell

**Affiliations:** 1 Dublin Dental University Hospital, Trinity College Dublin, Dublin, D02F859, Ireland; 2 The Irish Longitudinal Study on Ageing (TILDA), Trinity College Dublin, Dublin, D02PN40, Ireland; 3 Mercer's Institute for Successful Ageing, St James's Hospital, Dublin, Ireland

**Keywords:** ageing demographics, cohort studies, oral health, health surveys, public health dentistry

## Abstract

**Background**: Little is known about the current oral health status of adults in Ireland. The aim of this study was to assess the dental health of community-dwelling adults aged 50 years and over in Ireland and to compare the current status to previous national surveys.

**Methods**: The Irish Longitudinal Study on Ageing (TILDA) Wave 3 assessed the dental health of a subset of participants. Respondents attending for health assessments were offered a dental examination. The World Health Organization examination criteria were used.

**Results: **Of the 3111 people who were offered the dental assessment, 2525 were examined. Adults below 50 years of age and respondents whose dental health data were unavailable at the time of analysis were omitted, giving a final sample of 2504.  Among the dental assessment sample, 9.9% (249) were edentate. Of those aged 65 years and older, 15.6% were edentate while for the same age group 40.9% were edentate in the 2000-02 national survey. The mean number of teeth present in those aged 65 years or older was 14.9 for males and 14.2 for females, whereas in 2000-02 it was 9.9 and 7.4, respectively.  56.8% of the dentate sample had 10 or more tooth contacts. The mean DMFT of those aged 50 years or more was 18.5 and the Root Caries Index was 6.3. Between 2000-02 and 2014-5 (this study) in adults aged 65 years and over, the mean DMFT decreased from 25.9 to 20.1 and the Root Caries Index decreased from 11.6 to 9.1.

**Conclusion**: The results indicate the dental health of community-dwelling adults aged 50 years and over in Ireland improved since the previous survey of 2000-02. These improvements mean a change in the treatment needs of this age group and will require policy and service adjustments to meet these needs

## Introduction

The ageing population is one of the great challenges that will confront health services in developed countries in coming years. It is estimated that by 2050, the number of adults aged 60 years and older worldwide will increase from 901 million to 2.1 billion, and the adults called “oldest old” (80 years and over) will more than triple (125 to 434 million), from 2015 ^1^. In 2011, in Ireland, adults aged 65 years and over comprised 11.4% of the total population and this proportion is predicted to reach 22.4% in 2041. The proportion of adults aged 80 years or older is predicted to be 7.5% of the total population in 2046 ^2, 3^.

The ageing population faces the challenge of various chronic diseases including physical and mental health related conditions. Maintaining good oral health may also be a challenge in older people and poor oral health has been called a silent epidemic ^4^. Older adults may, as a result of reduced oral function, eat a poor quality diet and avoid social interaction and in this way poor oral health may adversely affect health and wellbeing. Similarly, loss of physical and cognitive function, along with increasing frailty, often result in less attention to oral self-care, while ill health or frailty may lead to reduced access to the professional care that is needed to maintain oral function.

Dental caries and periodontal disease are the most common chronic oral diseases affecting the adult population in Ireland and worldwide ^5, 6^, and the loss of natural teeth is considered to be a key indicator of poor oral health in older people ^7^. Although the complete loss of teeth with age has reduced dramatically in recent years, most oral health surveys have reported an age-related deterioration in oral health including an increase in tooth loss, poor periodontal health and increased tooth wear ^5, 8, 9^. The maintenance of a minimum number of functioning teeth, good periodontal health, absence of active dental caries and control of tooth wear are general indicators of satisfactory dental health in older adults. 

In Ireland, the last national survey of the oral health status of adults was undertaken in 2000–02, so up-to-date information reflecting the current oral health status of adults is lacking. This makes it difficult to assess the needs of the population and to design services that maintain the oral health of older adults. Wave 3 of The Irish Longitudinal Study on Ageing (TILDA) provided an opportunity to include a dental assessment of community dwelling adults aged 50 years and over ^10^. The aim of this assessment was to provide an up-to-date picture of the dental health of older adults, and to compare the current data with previous data from Irish and international studies.

## Methods

### Ethical considerations and consent

Ethical approval for this study was obtained from the Trinity College Dublin Faculty of Health Sciences Research Ethics Committee and participants provided written informed consent before the health assessment.

### Study design

TILDA is a large-scale, nationally representative, comprehensive cohort study on ageing in Ireland. It was started in 2009 and at the time of writing had completed its fourth wave of data collection ^11^. The TILDA cohort consists of randomly selected community-dwelling adults aged 50 years and over, although partners or spouses of any age can also participate. There are three modes of data collection, a computer aided personal interview (CAPI), self-completion questionnaire (SCQ) and health assessments ^10^.

In Wave 3 (March 2014 – December 2015), for the first time in the TILDA study a dental assessment was included as part of the health assessment conducted in the TILDA centre in Trinity College Dublin. The dental sample was a non-random convenience sample. As a dentist was not available during the full TILDA centre opening hours, only those participants who completed a health assessment during the dentist’s hours were invited to have the dental assessment; there were no other exclusion criteria. The respondents attending the TILDA centre in Dublin came from all over the country. Periodontal probing was omitted from the assessment of respondents at risk from bacteraemia. The examination criteria used in this study were the same as those used in previous Irish national oral health surveys and similar to those recommended by WHO ^5, 12^. The examiners were trained by an experienced examiner (‘gold standard’--JMcL) from previous studies. A total of one trainer (‘gold standard’) and four assessors (including AN, BOC) completed the data collection. As the dental assessment was at the end of the health centre assessment (approximately three hours long), a maximum of 10 minutes was allocated for it. Because of the time constraint, it was not possible to perform duplicate examinations during the data collection. During the pilot phase, a calibration exercise was performed on non-participant volunteers, followed by dual assessment of study participants until any discrepancies between the trainer and assessors were resolved. For the dental examination, standardised equipment consisted of a dental chair with floor mounted Daray LED examination light (Model- XL200 LED examination light, 12–30v/5.8–8.2w), standard plane dental mirror and WHO recommended Community Periodontal Index of Treatment Need (CPITN) probe-E ^12^. Methods and equipment were the same for all examinations. Standard cross infection control measures were followed during all examinations.

All respondents attending for a health assessment were invited to participate in the OHA while an examining dentist was present. The data collected was; number of natural teeth, use of dentures, CPITN on index teeth, coronal caries at cavitation level and visual level (WHO and British Association for the Study of Community Dentistry-BASCD) ^13^, Root Caries Index of Katz ^14^, coronal tooth wear into dentine ^15^ and tooth contacts between maxillary and mandibular teeth in the Maximum Intercuspal Position (MIP) ^16, 17^. All criteria were based on visual examination and tactile sensing methods using a CPITN probe and no radiographs were taken. Data from the assessment was written on a paper form and then entered on a laptop computer and uploaded to the TILDA database.

For the purpose of the CPITN examination, the mouth was divided into sextants and the highest (worst) score in each sextant was recorded as the sextant score. The scores were; no disease (H), bleeding on examination (B), supra or sub gingival calculus present (C), pocket depth up to 4-5mm (P1), pocket depth >6mm (P2) and if no teeth were present in a sextant/unable to record (X) ^12^.

Tooth contacts in the Maximal Intercuspal Position (MIP) were recorded to evaluate the functional dentition and the need for replacement of teeth ^16, 18– 21^. To achieve MIP, participants were asked to swallow and keep their teeth closed together-- the number of mandibular occlusal units in contact with maxillary teeth was counted. An occlusal unit was considered to be a single anterior tooth or premolar, or half a molar tooth (mesial or distal) ^21^. The percentages of dentate adults with fewer than 10 contacts, and 10 contacts or more, by age group and gender were calculated. Ten tooth contacts indicate approximately 20 teeth in occlusion, which is considered to be a minimal functioning dentition ^21^.

The presence of root caries was recorded in all respondents and the Root Caries Index (RCI) was calculated among the dentate adults who had exposed roots due to gingival recession, as was done in previous Irish national surveys of adults ^14^. This index gives the proportion of exposed roots with caries or restorations due to caries (RCI = mean decayed and filled roots /mean exposed roots %) in the population with exposed roots. Decayed and filled roots were recorded at tooth level rather than surface level, and so RCI was also calculated at tooth level.

Tooth wear was recorded by visual examination. The Bardsley tooth wear index was used in this study to record coronal tooth wear into dentine ^15^. The mouth was divided into sextants and each sextant was individually scored. Tooth wear was recorded as; no wear, exposed dentine comprised <1/3 of worst surface of a tooth, exposed dentine comprised >1/3 of worst surface of a tooth, or the sextant was excluded, as no teeth were present in the sextant or unable to record a score. The worst tooth in a sextant was recorded as a sextant score. The highest score of dentine wear per person was recorded as a person’s tooth wear level.

During the statistical analysis, some dental health indicators were calculated for the full dental assessment sample (denture wear, number of teeth and mean number of decayed, missing or filled teeth (DMFT)) and other indicators (tooth contacts, RCI and periodontal health) were calculated for the dentate sample only, resulting in two bases for results. “base edentate/dentate” means the statistical analysis involved the full sample, including edentate and dentate respondents, whereas “base dentate” means statistical analysis was run only on the dentate sample.

TILDA is subject to the legislation under the Data Protection Act 1988 and the Data Protection (Amendment) Act 2003. All data protection protocols were followed during collection, processing, analysis and reporting on data ^22^. Data analysis followed completion of data cleaning by accessing a TILDA hot desk at the TILDA research centre in Trinity College. STATA software (Stata 14.1 Stata Corp LLC Texas USA) was used for data analysis.

## Results

### Dental health assessment sample selection

The study sample was a sub-sample of the respondents who attended a TILDA health centre assessment at Wave 3. A total of 4309 respondents attended for the health assessment, of whom an opportunistic sample of 3111 (72.2%) were invited to have the dental assessment, and of these 2525 (81.1%) agreed to the assessment. Those aged less than 50 years (n=17) were omitted from this analysis. The full dental sample consisted of 2508 respondents, however, as the data for 4 of these respondents was not available at the time of the analysis, the results reported here are for 2504 respondents ^11^.

### Dental health assessment sample vs TILDA sample (population sample)

The TILDA cohort is a nationally representative sample of adults aged 50 years and over (a population sample). However, the dental assessment was completed on a convenience sample of the TILDA cohort. Table 1 below reports the characteristics of the dental sample and a two-sample proportion test (Z test) for comparisons of the characteristics between the dental sample and the Wave 3 TILDA cohort. For the purpose of comparison between the different samples, 10 variables with 23 categories were selected. There was no difference between the samples (dental and TILDA) in 8 out of the 23 categories.

**Table 1.** Comparison of the dental assessment sample with the TILDA sample by a two-sample proportion test (Z test). P values less than 0.05 are shown in bold.

Characteristic	Oral Health Assessment Sample (n=2508) n (%)	Population (TILDA sample) (n=6618) n (%)	Hypothesis test of proportions Population (TILDA) vs OHA
Age group			**P value**
50–64 years	1219 (48.6)	3036 (45.9)	**0.0196**
65–74 years	918 (36.6)	2110 (31.9)	**< 0.001**
≥75 years	371 (14.8)	1472 (22.2)	**< 0.001**
Female	1386 (55.3)	3679 (55.6)	0.7786
Education level			
Primary	478 (19.1)	1737 (26.3)	**< 0.001**
Secondary	1005 (40.1)	2610 (39.4)	0.5805
Tertiary/higher	1024 (40.8)	2269 (34.3)	**< 0.001**
Marital status			
Married	1889 (75.3)	4573 (69.1)	**< 0.001**
Never married	168 (6.7)	562 (8.5)	**0.0048**
Separated/ divorced	192 (7.7)	469 (7.1)	0.3494
Widowed	259 (10.3)	1014 (15.3)	**< 0.001**
Locality			
Dublin	681 (27.1)	1592 (24.1)	**0.0023**
Other urban	661 (26.4)	1840 (27.8)	0.1664
Rural	1166 (46.5)	3186 (48.1)	0.1588
Grew up in rural area	1440 (57.4)	3891 (58.8)	0.2331
Never lived abroad	669 (26.7)	1534 (23.2)	**0.0005**
Current or former smoker	1302 (51.9)	3609 (54.5)	**0.0251**
No health insurance or medical card	209 (8.3)	591 (8.9)	0.3681
Self-reported health			
Excellent	394 (15.7)	921 (14.2)	**0.0294**
Very good	897 (35.8)	2169 (33.4)	**0.0069**
Good	851 (34.0)	2227 (34.2)	0.8001
Fair	312 (12.4)	970 (14.9)	**0.0065**
Poor	52 (2.1)	215 (3.3)	**0.0029**

In summary, the dental sample was younger, more respondents were married, they were better educated, with good to excellent self-rated general health and more likely to be living in Dublin than the full TILDA sample.

### Dental health assessment sample description

For the analysis, the dental sample was stratified into three age groups as recommended by the WHO ^12^; 50–64 years old, 65–74 years old and 75 years and over (see Table 2 for gender breakdown). Almost half of the sample was aged 50–64 years, with 14.8% aged 75 years and over. Overall, the dental sample consisted of more females than males (55.3% vs 44.7%), but this trend was less marked in older respondents.

**Table 2.** The dental assessment sample by gender and age group (n=2504).

Gender	Age groups			Total
	50–64 years	65–74 years	75 years and over	
**Male**	511	422	187	1120
	42.0%	46.1%	50.5%	44.7%
**Female**	707	494	183	1384
	58.0%	53.9%	49.5%	55.3%
**Total Row %**	1218	916	370	2504
	48.6%	36.6%	14.8%	100%

### Dentate/edentate proportion

The adults with at least one natural tooth present were recorded as dentate. Table 3 shows that overall, 9.9% of the sample was edentate (no teeth).

**Table 3.** Number and percentage of edentate/dentate sample by age group and gender (Base edentate/dentate, n=2504).

Age group	50–64 years		65–74 years		75 years & over		Total
Gender	Male	Female	Male	Female	Male	Female	
**Edentate**	15	33	36	71	39	55	249
	2.9%	4.7%	8.5%	14.4%	20.9%	30.1%	9.9%
**Dentate**	496	674	386	423	148	128	2255
	97.1%	95.3%	91.5%	85.6%	79.1%	69.9%	90.1%

The proportion of edentate adults was higher in the older age group, and more females were edentate than males in all age groups. Overall, 9.9% of respondents were completely edentate, 14.0% were edentate in the upper arch only, and 19% were edentate in the lower arch only.

### Denture wear

Table 4 shows that 46.9% of the sample had some type of removable denture. While 9.9% of the sample was edentate, only 9.1% was wearing complete dentures for both the upper and lower arches.

**Table 4.** The proportion of denture wearers by age group and type of denture (Base edentate/dentate, n=2504).

Age group	No upper or lower denture		Complete upper and lower dentures		Complete upper and partial lower dentures		Complete upper denture only		All other combinations of complete and partial dentures		Total
	n	%	n	%	n	%	n	%	n	%	n
**50–64** **years**	838	68.8	42	3.5	10	0.8	41	3.4	287	23.6	1,218
**65–74** **years**	392	42.8	98	10.7	51	5.6	67	7.3	308	33.6	916
**75 years** **and over**	100	27.0	88	23.8	24	6.5	41	11.1	117	31.6	370
**Total**	1,330	53.1	228	9.1	85	3.4	149	5.9	712	28.4	2504

The percentage of adults wearing dentures was higher in the oldest age group irrespective of the type of denture. In those aged 75 years and over, 73% wore dentures and 24% wore complete dentures.

### Number of teeth

**Figure 1.**
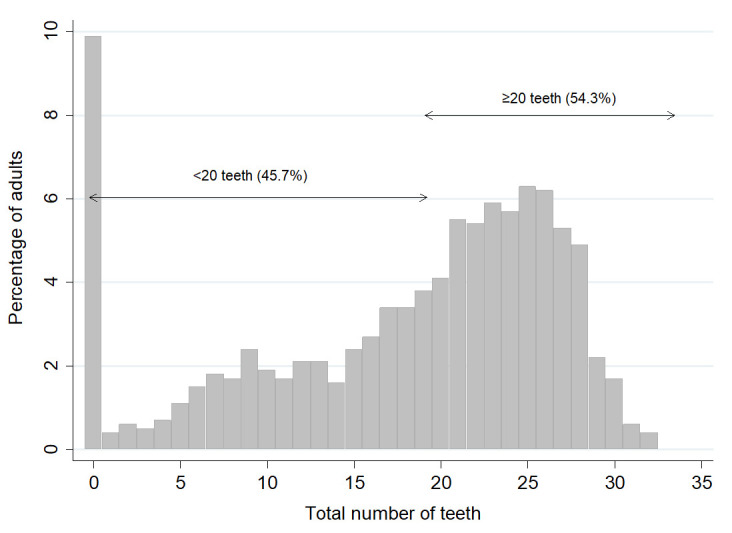
Percentage of adults by total number of teeth and the proportion with <20 teeth and ≥ 20 teeth. (Base edentate/dentate, n=2504). Mean number of teeth was 17.9, SD = 8.9, Median=21.

Figure 1 shows the frequency distribution of the number of teeth present in the dental sample. It shows that 9.9% of adults had no teeth, and 54.3% of adults had 20 or more teeth. When the results in Table 5 are compared, it can be observed that although a higher proportion of females were edentate, females in the youngest age group, who were dentate, had a similar mean number of teeth to males (21.5 vs 21.1). This suggests that the higher tooth loss in older women in Ireland may be reversing, although this would need to be substantiated by a longitudinal study.

**Table 5.** Mean number of teeth per person by age group and gender (Base edentate/dentate, n=2504).

Age groups	Male	Female	Total
**50–64 years**	21.1	21.5	21.3
**65–74 years**	16.3	15.4	15.8
**75 years & over**	11.9	11.1	11.5
**Total**	17.7	17.9	17.9

### Tooth contacts

**Figure 2.**
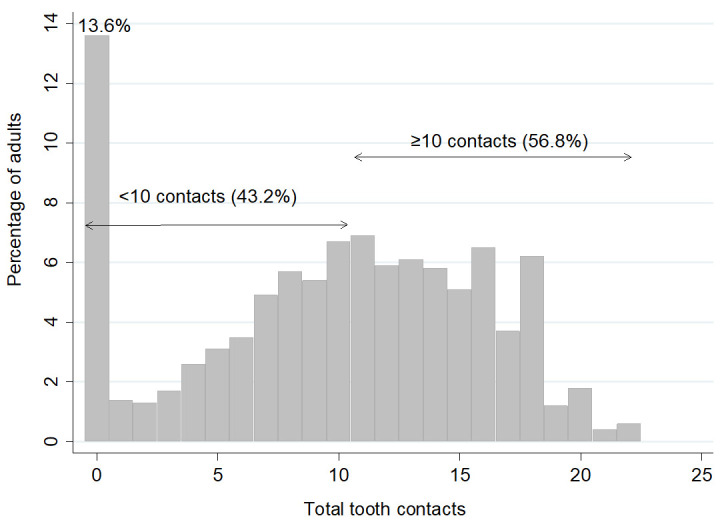
Percentage of adults by total number of tooth contacts and with <10 tooth contacts and ≥10 contacts (Base dentate, n=2255). Mean 9.9, SD= 5.9, Median= 11.

The percentage of dentate adults with fewer than 10 occlusal contacts, and 10 contacts or more, by age group and gender was calculated. Figure 2 shows that 56.8% of the dentate sample had 10 or more tooth contacts. Notably, 13.6% of dentate adults had no contacts; these adults were edentate in one arch, wearing dentures, had cross bites, teeth not in contact with other teeth, or just roots remaining. Table 6 shows that the proportion of dentate adults with 10 or more contacts was less in the oldest age group and was higher in females, although the gender difference narrowed in the older age groups.

**Table 6.** Number and percentage of adults with fewer than 10 tooth contacts, and equal to or more than 10 tooth contacts, by age group and gender (Base dentate, n=2255).

Age group	50–64 years		65–74 years		75 years & over		Total
Gender	M	F	M	F	M	F	
**<10 Contacts**	172	179	214	225	101	84	975
	34.7%	26.6%	55.4%	53.2%	68.2%	65.6%	43.25%
**≥ 10 Contacts**	324	495	172	198	47	44	1280
	65.3%	73.4%	44.6%	46.8%	31.8%	34.4%	56.75%

### Decayed, missing and filled teeth (DMFT)

Dental caries was recorded at cavitation (DMFT-c) and at visual caries level (DMFT-v). As the results indicated a difference of 0.1 between DMFT-c and DMFT-v (DMFT-c= 18.5, DMFT-v =18.6), it was decided to only report DMFT-c.

During the dental assessment it was possible to identify that some teeth were missing for reasons other than dental caries. These were missing premolars with no residual spaces (orthodontic extractions or congenital absence), third molars extracted due to impaction and teeth lost due to trauma. Where there was certainty about the reasons for loss these teeth they were recorded as missing for other reasons and not counted in the M component of DMFT. Where it was unclear, or there was doubt about the reasons for tooth loss, these teeth were recorded as missing due to caries. Similarly, in the edentate group third molar teeth were not recorded as missing due to caries. For this reason, the maximum DMFT score for edentate adults is shown as 28 but for dentate adults is 32.

**Figure 3.**
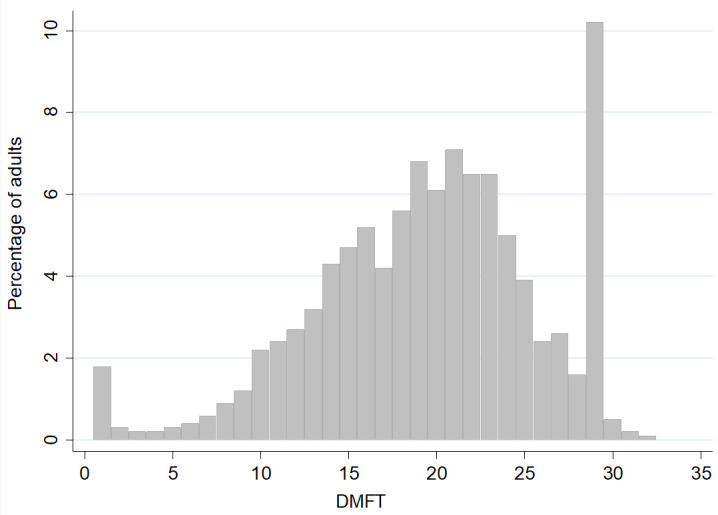
Frequency distribution of the percentage of adults aged 50 years and over by DMFT score (Base edentate/dentate, n=2504). Mean= 18.5, SD= 6.3, Median=19.

**Figure 4.**
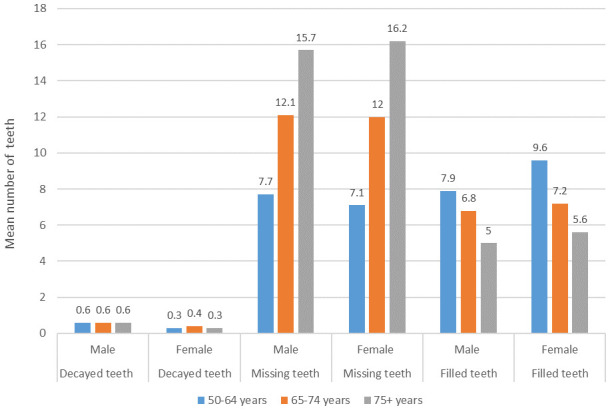
Mean decayed, missing and filled teeth components of DMFT by age group and gender (Base edentate/dentate, n=2504).

Figure 3 shows that overall, 10.2% of adults had a DMFT score of 28, which includes the 9.9% who were edentate. The DMFT scores are negatively skewed with a greater percentage of adults having high values for DMFT. Only 1.8% of adults had a DMFT score of 1. Mean DMFT values by age group and gender are reported in Table 7. Overall, females had a slightly higher mean DMFT (0.3) score than males, and this gender difference was present among all three age groups. Figure 4 shows the contribution of the decayed teeth, missing teeth, and filled teeth components of DMFT by age group and gender.

**Table 7.** Mean DMFT level by age group and gender (Base edentate/dentate, n=2504).

Age groups	Male	Female	Total DMFT
**50–64 years**	16.4	17.0	16.7
**65–74 years**	19.4	19.6	19.5
**75 years & over**	21.3	22.0	21.6
**Total**	18.3	18.6	18.5

Of the total mean DMFT of 18.5, the contribution of decayed teeth was 0.6 in males and 0.3 in females, and was almost the same in all age groups. When compared to men, women in all age groups had fewer decayed teeth, more filled teeth, and women aged 75 years and over had more missing teeth, which suggests that women access treatment more. It is notable that in the youngest age group, the proportion of missing teeth was much lower than in the two older age groups, with corresponding higher proportions of filled teeth and the same proportion of decayed teeth. It remains to be seen whether this younger cohort can maintain more of their natural teeth as they age, as this would represent a major shift in the dental health of older adults in Ireland.

### Root caries

Root caries is reported as the mean number of decayed and filled roots as a proportion of the mean number of roots with recession ( Table 8). The RCI (4.3 vs 10.2) and the mean number of decayed/filled roots (0.5 vs 1.1) was higher, and the mean number of exposed roots (11.1 vs 10.3) was lower, in the oldest age group. in the lower mean number of exposed roots may be due to having fewer teeth with increasing age. Females aged less than 75 years had higher levels of root caries than males, but those aged 75 years and over had slightly lower levels than males.

**Table 8.** Mean number of exposed roots, mean decayed/filled roots and Root Caries Index (RCI) by age group and gender (Base dentate, n=2255).

Age groups	Mean decayed/filled roots (DFR)			Mean exposed roots (ER)			RCI		Total RCI
	Male	Female	Total	Male	Female	Total	Male	Female	
**50–64 years**	0.4	0.5	0.5	11.6	10.7	11.1	3.6	4.9	4.3
**65–74 years**	0.8	0.9	0.9	11.1	10.7	10.8	7.2	8.3	7.8
**75 years & over**	1.1	1.0	1.1	10.1	10.6	10.3	10.6	9.8	10.2

### Periodontal health

The CPITN was used for the periodontal health assessment. The severity of periodontal disease was reported by the maximum CPITN score per person, and the extent as the mean number of sextants affected by the different scores for the dentate adults. Table 9 shows that the proportion of men and women with completely healthy periodontal tissues or bleeding gingivae or deep pockets was low, at less than 5%, in all age groups. The majority of respondents needed simple treatment for calculus and shallow pockets. The gender differences were small, with females tending to have better periodontal health status.

**Table 9.** Percentage and total number of adults with a maximum CPITN score of H (healthy), B (bleeding), C (calculus), P1 (shallow pocket), P2 (Deep pocket) and X (missing sextant) by age groups and gender (Base-Dentate n=2255).

Age groups	H%		B%		C%		P1%		P2%		X%		Total n
	M	F	M	F	M	F	M	F	M	F	M	F	
**50–64 years**	1.4	3.9	1.1	2.0	10.9	18.4	24.3	30.6	4.1	2.5	0.6	0.3	1170
**65–74 years**	2.4	3.5	0.9	2.7	14.3	19.3	24.9	23.9	2.7	2.2	2.6	0.7	809
**75 years and over**	3.6	4.7	3.3	1.8	15.9	19.9	22.1	17.4	2.5	1.5	6.2	1.1	276
**Total n**	45	86	29	50	288	426	546	599	77	51	45	13	2255

Table 10 shows the mean number of sextants per person affected by the different CPITN scores, stratified by age group and gender. The mean number of sextants with deep pockets is low (0.1–0.2). With respect to shallow periodontal pockets, the mean number of sextants affected was between 0.8 and 1.5, which suggests that pockets were not very extensive in this sample. The results from these two tables indicate that the periodontal treatment needs of this sample were neither complex nor extensive.

**Table 10.** Mean number of sextants per person affected by different CPITN score: H (healthy), B (bleeding), C (calculus), P1 (shallow pocket), P2 (Deep pocket) and X (missing sextant) among dentate sample by age group and gender (Base dentate, n=2255).

Age groups	H		B		C		P1		P2		X	
	M	F	M	F	M	F	M	F	M	F	M	F
**50–64 years**	1.6	2.2	0.6	0.7	1.4	1.3	1.5	1.2	0.2	0.1	0.7	0.5
**65–74 years**	1.4	1.9	0.5	0.5	1.4	1.3	1.1	0.8	0.1	0.1	1.5	1.4
**75 years & over**	1.4	1.7	0.4	0.4	1.1	1.2	0.9	0.8	0.1	0.1	2.2	1.8

### Tooth wear

Table 11 shows that in all age groups, fewer than 7% of respondents had no wear into dentine, while 50.6% had wear into dentine on less than one third of the worst surface. In all three age groups, there was more severe wear in males than females. In the age groups included in this study, some tooth wear would be considered to be physiological and for this reason, the low percentage with no wear is not unexpected.

**Table 11.** Percentage of adults with no tooth wear, dentine exposed less than 1/3 of worst surface, dentine exposed more than 1/3 of worst surface, or sextant excluded, among the dentate sample by age group and gender (Base dentate, n=2255).

Age groups	No wear		Wear <1/3 of dentine		Wear >1/3 of dentine		Excluded		Total
Gender	M	F	M	F	M	F	M	F	n
**50–64 years**	3.3	6.9	20.3	32.3	18.5	17.7	0.3	0.7	1170
**65–74 years**	3.3	5.4	20.2	28.1	23.9	18.4	0.5	0.4	809
**75 years and** **over**	2.5	4.7	23.9	25.4	26.1	16.3	1.1	0	276
**Total n**	73	138	465	675	482	401	10	11	2255

## Discussion

In Ireland, there have been considerable improvements in the oral health status of adults from the previous Irish oral health surveys conducted in 1989–1990 ^23^ and 2000–2002 ^5^. A summary of the principal oral health indicators in older adults in Ireland, from the last three surveys, is shown in Table 12. For national and international comparisons DMFT is reported by the WHO method of calculation (including teeth missing for all reasons).

**Table 12.** Changes in edentulism, mean number of teeth, DMFT (WHO calculation) and RCI among adults aged 65 years and over in Ireland from 1989–90 ^23^ to 2000–02 ^5^ and in the current study, 2014–15.

Oral health indicators	Examination Year	Male	Female	Total
**% Edentate**	**2014–15**	12.3	18.6	15.6
	**2000–02**	34.6	45.6	40.9
	**1989–90**	33	61	48
**Mean number of teeth**	**2014–15**	14.9	14.2	14.6
	**2000–02**	9.9	7.4	8.5
	**1989–90**	10.1	4.9	7.3
**Mean DMFT**	**2014–15**	23.9	24.8	24.4
	**2000–02**	24.8	27	25.9
	**1989–90**	25.6	28.8	27.3
**RCI**	**2014–15**	9.1	9.1	9.1
	**2000–02**	12.7	10.6	11.6
	**1989–90**	20.9	14.9	18.5

***Edentulism.*** Table 12 indicates that in Ireland, among adults aged 65 years and over, the prevalence of edentulism has reduced by more than two-thirds from 1989–90 to 2014–15 and most of that decrease has occurred since 2000–02. Though the prevalence of edentulism was reduced more dramatically in females than males, in 2014–5 the prevalence was still about 50% higher in females. A comparison of the Irish and international prevalence of edentulism is shown in Table 13.

There are similar age differences in the prevalence of edentulism in different countries, although the age groups and dates of the studies vary. For example, in the age group 75 years or older, the TILDA dental sample in 2014–15 had a similar prevalence of edentulism to the USA in 2012. For the group aged 65–74 years, edentulism in the TILDA dental sample (11.7% in 2015) and in the UK (15.0% in 2009) are comparable taking account of the time difference between the studies.

**Table 13.** Percentage edentulism by age groups, country and examination year.

Country	Year of Examination	Age group	% Edentate
** Ireland**	**2014–15**	50–64 years 65–74 years 75 years and over	3.9 11.7 25.4
**UK ^24^**	**2009**	55–64 years 65–74 years 75–84 years 85 years and over	5 15 30 47
**USA ^25^**	**2012**	65–74 years 75 years and over	13 25.8
**Australia ^26^**	**2006**	55–74 years 75 years and over	13.9 35.7
**New Zealand ^27^**	**2009**	55–64 years 65–74 years 75 years and over	14.5 29.6 39.6

***Mean number of teeth.*** Table 12 shows that in Ireland there has been a doubling in the mean number of teeth in adults aged 65 years and older from 1989–90 to 2014–15 (7.3 to 14.6). This trend in females is particularly positive where the mean number of teeth almost doubled between 2000-02 and 2014–15. Although mean number of teeth is a crude measure of oral health status, as it gives no indication of the condition of these teeth, it is nonetheless a positive trend that natural teeth have been retained rather than extracted.

Table 14 shows that adults in Ireland, aged 65–74 years, and 75 years and over, had a lower mean number of teeth than the UK, New Zealand and Australia, despite the fact that the studies in these countries were completed 5–10 years before the TILDA study. Together, these findings suggest that in Ireland, fewer adults above 65 years may be completely edentate than in this group of countries, but the mean number of teeth per person is also less than the UK, Australia and New Zealand.

**Table 14.** Mean number of teeth per person by age groups, country and year of examination among Dentate Sample.

Country	Year of examination	Age group	Mean number of teeth
**Ireland**	**2014–15**	50–64 years 65–74 years 75 years and over	22.2 17.9 15.4
**UK ^24^**	**2009**	55–64 years 65–74 years 75–84 years 85 years and over	23.2 20.9 17.1 14.0
**Canada (CHMS) ^28^**	**2007–09**	40–59 years 60–79 years	24.1 19.4
**New Zealand ^27^**	**2009**	55–64 years 65–74 years 75 years and over	24.0 19.7 18.1
**Australia ^26^**	**2004–06**	65–74 years 75 years and over	21.8 17.9

***Dental caries.*** There was a decrease in mean DMFT from 1989–90 to 2014–15, for those aged 65 years and older in Ireland (27.3 vs 24.4), as shown in Table 12. It is notable that most of this decrease in DMFT occurred since 2000–02. From 1989-90 to 2014–15, the gender difference in mean DMFT also reduced from 3.2 to 0.2.

These findings, along with the doubling of the mean number of teeth ( Table 12), suggest that adults over 65 years are not only keeping more of their teeth, but these teeth are in a healthier state. It also appears that these adults have accessed dental care which is more oriented to the maintenance of teeth than in the past.

Table 12 also indicates that the Root Caries Index reduced by more than half in Ireland from 1989–90 to 2014–15 (18.5 vs 9.1). The gender difference for RCI was reduced from 6 in 1989–90 to 0 in 2014–15. This is an encouraging finding, as it was thought that the prevalence of root caries in older people might increase with increased retention of natural teeth and a tendency to lose periodontal attachment with age, resulting in more exposed root surfaces that are vulnerable to the development of root caries.

Comparison of the mean DMFT among the TILDA dental sample and other countries is shown in Table 15. It should be noted that in both the Australian and New Zealand surveys these mean DMFT values were for the dentate population, as edentate people were excluded from the oral health assessments.

**Table 15.** Mean DMFT (WHO method) by age groups, country and examination year.

Country	Year of Examination	Age group	Mean DMFT
** Ireland**	**2014–15**	50–64 years 65–74 years 75 years and over	20.1 23.7 26.2
New Zealand * ^27^	**2009**	55–64 years 65–74 years 75 years and over	21.7 24.2 24.8
Australia * ^26^	**2004–06**	55–64 years 65–74 years 75 years and over	21.7 23.2 24.6

*Dentate sample only

The TILDA dental sample had a lower mean DMFT than data from New Zealand and Australia, however, these studies took place 5–10 years before the Irish study which might account for some of this difference in mean DMFT. The inclusion of the edentate group in the TILDA calculation of mean DMFT would suggest that the difference in mean DMFT between Ireland and Australia and New Zealand may in fact be greater than it appears in this table.

***Periodontal health.*** In Ireland, the changes over time in the proportion of adults aged 65 years and over with maximum CPITN scores from 2000–02 to 2014–15 are shown in Table 16. From 2000–02 to 2014–15, the proportion of adults with a CPITN score of ‘healthy’ had slightly reduced. There was a noticeable increase in the proportion of people with calculus and shallow periodontal pockets, but a substantial reduction in the proportion with deep periodontal pockets. It is also important to note the fall in the number of excluded sextants (X), which indicates that more sextants had the minimum number of teeth for the CPITN examination to be carried out. The increase in the proportion with calculus and shallow pockets probably also reflects increased tooth retention.

**Table 16.** Changes over time in percentage of adults with maximum value of CPITN-severity score of H (healthy), B (bleeding), C (calculus), P1 (shallow pocket), P2 (deep pocket) and X (missing sextant) among dentate adults 65 years and over in Ireland (Base dentate).

Year of examination	H	B	C	P1	P2	X	Total (n)
**2014–15**	6.6	4.0	35.1	46.4	4.7	4.3	1085
**2000–02**	6.9	3.6	29.5	37.6	12.0	10.1	390

Data on the health of the population is a key part of identifying needs, planning public health strategies and assessing the effectiveness of public health policies. This is especially true of oral health, which is sensitive to socioeconomic conditions, dietary trends, lifestyle and access to care. At the same time, most dental disease is preventable so there is the potential for improvement in oral health at relatively low cost ^29^. The last national survey of adult oral health in Ireland was conducted in 2000–02 and the results were published in 2007. With the implementation of a new national oral health policy underway it is critical to understand the current oral health status of older adults in Ireland. The TILDA study was a valuable opportunity to examine the oral health of a nationally representative cohort of older Irish adults: this cohort has been extensively characterised in terms of their physical and mental health, wellbeing, social interactions, and socioeconomic status.

The respondents who participated in the dental assessment were similar to the whole TILDA cohort in the key areas of gender, medical card status, urban-rural dwelling, and self-reported health status. The dental group had more respondents from Dublin, were younger, and were more likely to have tertiary/higher level education than the nationally representative TILDA cohort. Comparing this study to previous national surveys, the prevalence of edentulism has continued to decline rapidly in Irish adults, following the trend seen in many other English-speaking countries, though it was noted that older women are still more likely than men to have no natural teeth at all. The comparison also highlights economic, cultural, and historic differences between countries with respect to edentulism; for example New Zealand had a much higher prevalence of edentulism than the UK at the same time point, perhaps due to sociological reasons ^30^.

Having at least 20 natural teeth, or 10 pairs of contacting teeth, is considered a benchmark of a functional dentition, that is a dentition that will generally provide adequate functional capacity and may not require additional prosthetic teeth ^21^. This study found that 56.8% of the dental sample had 10 or more pairs of contacting teeth, though as with other variables, there was a marked difference between the younger and older age groups. This means that there is a much larger need for intervention in the form of replacement teeth among older adults in Ireland, though a report on this cohort found that people with fewer teeth were actually less likely to access dental services ^31^.

One of the concerns raised about maintaining oral health in older people is that, paradoxically, the retention of natural teeth incurs more ongoing care needs than a complete loss of teeth. Without adequate regular plaque control, a healthy diet and some professional dental care, natural teeth can be susceptible to caries and periodontal disease which may lead to pain, infection and loss of function. The susceptibility of older adults to dental disease may be heightened due to age-related periodontal recession and an increase in medications that cause dry mouth. In Ireland, adults who become frail, dependent, or live in residential care will often lose access to regular dental services. This study found a low level of untreated crown and root caries, and mostly mild periodontal disease, which suggest that independently-living older adults can maintain their oral health fairly well. Nonetheless, the level of root caries in those aged 75 years or older was double that of the younger age groups. The challenge will be to provide adequate care for the growing number of frail older people who, in the future, will have a far greater number of natural teeth.

This study made a broad assessment of dental status, using a methodology that allows for comparison to previous national oral health surveys and similar international studies. Other strengths of the study design were the large sample size and the extensive information collected during the CAPI, SCQ and health assessment. This will be extremely useful in further analysis that will link the objective measures of dental health with other health outcomes. The dental sample also had similar characteristics to the TILDA cohort, suggesting that the findings should be representative of the overall sample. However, the study design had some limitations. The participants in the dental assessment were recruited from those attending a health centre assessment; participants who had a home-based assessment or did not complete a health assessment were older and had poorer general health indicators than those who did attend a health centre ^32^ so it is likely that the oral health status of these participants is also poorer than the dental sample. Therefore, while it was possible to generalise these results to the majority of the community-dwelling population aged 50 years and over, they may underestimate the prevalence and extent of oral health problems in adults with poorer health and those in residential care.

## Conclusions

Data from this study shows that there has been considerable improvement in the retention of teeth among community dwelling adults aged 50 years and over in Ireland, since previous Irish national surveys. Since 1989–90, the proportion of edentate adults aged 65 years and over has decreased by two-thirds, and the mean number of natural teeth has more than doubled. However, tooth loss was still common among older adults, posing a challenge for our health services. Currently, there is little service provision for dentate older adults, especially those who become frail or dependent, suggesting the need for a qualitative change in the oral care for older people in Ireland.

If generalised to the whole population, these findings suggest that Ireland has more slowly followed international trends for improvement in the oral health status of older adults, observed in other developed countries like the UK, USA, Australia and New Zealand. These trends are very positive but they indicate a requirement for more maintenance care, restorative and periodontal treatment, and less need for complete dentures than previously. The findings of this study should provide a valuable resource for oral health policy and planning of oral health services for older people in Ireland.

## Data availability

The data presented in this report was collected during Wave 3 of TILDA. Wave 3 data is available from the Irish Social Science Data Archive (ISSDA):

ISSDA: Dataset 1. The Irish Longitudinal Study on Ageing (TILDA) Wave 3, 2014–2015. Study number: 0053-04. www.ucd.ie/issda/data/tilda/wave3 ^33^.

### Accessing the data

To access the data, please complete a ISSDA Data Request Form for Research Purposes, sign it, and send it to ISSDA by email ( issda@ucd.ie).

For teaching purposes, please complete the ISSDA Data Request Form for Teaching Purposes, and follow the procedures, as above. Teaching requests are approved on a once-off module/workshop basis. Subsequent occurrences of the module/workshop require a new teaching request form.

Data will be disseminated on receipt of a fully completed, signed form. Requests to access the dental assessment data should be made directly to TILDA ( tilda@tcd.ie)

### Consent

Ethical approval for this study was obtained from the Faculty of Health Sciences Research Ethics Committee in Trinity College Dublin and participants provided written informed consent before the health assessment.
