# Supplementary material for: Dental health status of community-dwelling adults aged 50 years and over in Ireland. A cross-sectional analysis of the Wave 3 TILDA cohort
Source: HRB Open Res. 2022 Feb 8;1:26. Originally published 2018 Dec 14. [Version 3] doi: 10.12688/hrbopenres.12891.3 (PMC8829095; doi:10.12688/hrbopenres.12891.3)
Supplement: Peer review history

# Peer review: Reviewer response for version 3

**DOI:** 10.21956/hrbopenres.14705.r31432

**Published:** 2022-02-09

**Authors:** W. Murray Thomson

**Affiliations:** 1 Department of Oral Sciences, Sir John Walsh Research Institute, Faculty of Dentistry, University of Otago, Dunedin, New Zealand

Congratulations to the authors for a fine report. I have no further comments or suggestions.

Is the work clearly and accurately presented and does it cite the current literature?

Partly

If applicable, is the statistical analysis and its interpretation appropriate?

Partly

Are all the source data underlying the results available to ensure full reproducibility?

No source data required

Is the study design appropriate and is the work technically sound?

Yes

Are the conclusions drawn adequately supported by the results?

Partly

Are sufficient details of methods and analysis provided to allow replication by others?

Partly

Reviewer Expertise:

Dental epidemiology, gerodontology, health services research

I confirm that I have read this submission and believe that I have an appropriate level of expertise to confirm that it is of an acceptable scientific standard.

# Peer review: Reviewer response for version 2

**DOI:** 10.21956/hrbopenres.14597.r30347

**Published:** 2021-09-24

**Authors:** W. Murray Thomson

**Affiliations:** 1 Department of Oral Sciences, Sir John Walsh Research Institute, Faculty of Dentistry, University of Otago, Dunedin, New Zealand

Overall, the paper is much improved – well done! I particularly like how the Introduction has improved.

 I have only minor points for the authors:

- In the Discussion, “compared to” is still used in a number of places – surely “than” is a better word to use than that awful phrase. In the 3 ^rd^ Discussion paragraph, the term “trend” is misused – “similar trends in the prevalence of edentulism with age” should read “similar age differences in edentulism prevalence”.
- Finally, the authors point out that it is difficult to compare their survey’s mean number of teeth with estimates from other national surveys because the latter have not included edentulous people in the denominator. Shouldn’t that be a very strong signal to them that one does not do that? That an estimate for the mean number of teeth should be just for the dentate sample?? I think they need to recalculate their mean number of teeth accordingly so that they are consistent with standard practice.

Is the work clearly and accurately presented and does it cite the current literature?

Partly

If applicable, is the statistical analysis and its interpretation appropriate?

Partly

Are all the source data underlying the results available to ensure full reproducibility?

No source data required

Is the study design appropriate and is the work technically sound?

Yes

Are the conclusions drawn adequately supported by the results?

Partly

Are sufficient details of methods and analysis provided to allow replication by others?

Partly

Reviewer Expertise:

Dental epidemiology, gerodontology, health services research

I confirm that I have read this submission and believe that I have an appropriate level of expertise to confirm that it is of an acceptable scientific standard, however I have significant reservations, as outlined above.

# Response

**Published:** 2022-01-05

**Authors:** Brian O'Connell

**Affiliations:** Dublin Dental University Hospital, Ireland

- In the Discussion, “compared to” is still used in a number of places – surely “than” is a better word to use than that awful phrase. In the 3 ^rd^ Discussion paragraph, the term “trend” is misused – “similar trends in the prevalence of edentulism with age” should read “similar age differences in edentulism prevalence”.

Thank you for spotting this. The recommended changes have been made in the attached revised version.

  

- Finally, the authors point out that it is difficult to compare their survey’s mean number of teeth with estimates from other national surveys because the latter have not included edentulous people in the denominator. Shouldn’t that be a very strong signal to them that one does not do that? That an estimate for the mean number of teeth should be just for the dentate sample?? I think they need to recalculate their mean number of teeth accordingly so that they are consistent with standard practice.

In both Irish adult surveys the mean number of teeth was calculated using the dentate and edentate samples. For that reason we calculated the mean number of teeth by the same method and reported this in the results and for national comparison (Table 12). However, as per your suggestion for international comparisons the mean number of teeth was calculated in the dentate sample only and the international comparison (Table 14) was updated.

# Peer review: Reviewer response for version 1

**DOI:** 10.21956/hrbopenres.13960.r26445

**Published:** 2019-01-31

**Authors:** Roseann Mulligan

**Affiliations:** 1 Division of Dental Public Health and Pediatric Dentistry, Herman Ostrow School of Dentistry, University of Southern California, Los Angeles, CA, USA

**Overview**

 There have been three waves of The Irish Longitudinal Study on Ageing (TILDA). In this work, the third wave is described during which time oral examinations were completed for a subset of participants aged 50 and over. The World Health Organization examination criteria was used to assess oral health as well as a number of previously used indices. Data on a variety of oral health indicators including number of retained teeth, number in occlusion, presence/absence of prostheses, caries, including root caries and periodontal disease indicators are presented and the results compared to previous Irish studies and findings from studies in other countries using similar criteria.

**Overall comments:**

 There is a large amount of data of all types that was collected into this one paper. Typically the results of large studies are published in multiple papers that can orient the reader toward the findings in various discrete areas, such as edentulism, caries, periodontal findings, and allow discussion about each respective area. This paper has some of the characteristics of a monograph, displaying the results of all the data collected relating to all oral health findings and therefore the multitude of tables and figures is to be expected. I appreciate having all the OHA data in the TILDA displayed in one publication and suggest that the authors also provide an appendix of all the figures that one can readily negotiate when looking for a particular topic. However, unlike a monograph there is a lack of detailed  discussion of findings relative to data about each specific finding. Usually only one reference is mentioned per topic and therefore the richness and meaning of the findings as compared to that found in other studies is often not expanded or expounded upon.

**Comments on the Introduction:**

 While the sample size for the Oral Health Assessment (OHA) equaled 2504 individuals aged 50 and over, nearly ½ of the sample was made up of middle-aged adults from 50 to 64 year olds and  those aged 75 and older only accounted for 14.8% (N = 371) of the entire OHA substudy. In most developed nations, 50 year olds are not considered elderly nor are their oral health findings that different from other middle-agers. Since TILDA is a Longitudinal Study on Aging, I was disappointed not to find more data about older adults, with details highlighting those 65+ and older by decades. Clearly the small sample size for the 75+ aged population precluded the ability of the investigators to analyze and present data. Therefore the authors’ focus in the Introduction that brings to the readers’ attention the significant growth occurring in the 65 and older and 80 and older age groups is puzzling as it whets the appetite to learn new findings that are not in fact available or presented. I suggest rewriting most of the Introduction to remove this emphasis.

**Comments about Methodology:**

 If the goal was to achieve robust cell sizes of older adults for analytic purposes and to make policy recommendations, I wonder why an oversampling methodology of the oldest categories was not used. Since this study appears to be a convenience sample of the overall TILDA pool of subjects attending a Health Center in Dublin, I have concern about its ability to serve as a representation of the general population of Ireland, which appears to be an objective of TILDA. Should there be future TILDA with OHA components, over sampling would be valuable of older age groups and I would strongly recommend such a strategy. If a longitudinal study is planned for the future, the current middle aged and newly aged elderly of the OHA substudy of TILDA 3 could serve as a baseline population for another wave. Still the subject selection would need to be improved upon by including cohorts outside of Dublin, so that the results have true national representativeness.

**Comments on Results:**

 Displaying the large amounts of data is challenging, I can appreciate the authors attempts to include all of their findings in this manuscript. I find the tables much more helpful as they actually show the results with the sample sizes noted at the basic levels, and for the most part the data is not collapsed into large categories, thus making it easier to compare with other studies. For those who want to make future comparisons of this data to newer datasets, it will be the Tables that will provide the data cited. The Figures were more difficult to interpret even though visually more appealing with their display of comparative and trend data; they have less practicality or comparability. Figure 4 contains a highly pictorial and easy to grasp display of the separate components of the DMFT findings by three age adult categories. This is the figure I suggest the authors retain.

 In the Results section there are many comparisons between previous findings in earlier Irish studies, however no statistical analysis were completed to determine if these differences were significant. Table 17 highlights the difficulty in this approach with the comparisons between the 2000-02 and 2014-15 studies described in the narrative as “slightly” and “substantial” to describe differences rather than indicating on the table whether or not there are statistical differences between the two studies using convention p value cut-offs. 

 While much is made of the valuable opportunity the TILDA study presents due to its extensive record of physical, mental and socioeconomic data on participants, few correlations were drawn between the oral findings outside of gender and middle to early older age groups due to sampling inadequacies (recruited small sample size of 75 and older individuals and the limited sample collection site.) The opportunity to better characterize those variables associated with individuals with the greatest oral health needs was missed.

 Because there are so many tables, it is difficult to flip through different pages to find the table and narrative when they are not in proximity. While sometimes it is inevitable that tables and narratives are not on the same page, more effort needs to be made by the editor to place some of the smaller tables in close proximity to the relevant discussion.

**Comments on Discussion:**

 I suggest that the authors be more careful about making generalizations about cause. In the discussion of the RCI findings on page 13, the authors state without citation that the explanation for an expected increase in root caries would be: “ . . . because teeth tend to lose periodontal attachment with age and exposed root surfaces are vulnerable to the development of root caries.” While many would agree with the second statement, not all would agree with the first statement that periodontal disease as defined by attachment loss is an inevitable component of aging. Nor does this type of statement belong in the Results section for this discussion of potential causality should be covered in the Discussion. 

 I find statements such as, “The increase in the proportion with calculus and shallow pockets probably also reflects increased tooth retention.” If increasing tooth retention is aligned with calculus and shallow pockets, why not the other measured variables that trended in the same direction? If statistically significant comparisons were made and posted on the table, the readers would be able to determine if for example whether the increase in bleeding or the decrease in healthy tissue were significant? I again suggest that statistical analysis be run and the results indicated on the table so that readers can draw conclusions. While it might be difficult to compare results from various countries due to differing methodologies, where the CPITN was used in both Irish studies, one would expect some comparisons so that the importance or lack thereof could be appreciated.

 It is in the Discussion section that one learns of the difficulties and differences in the OHA sub sample. The population sampled should have been described in the Methods section, not in the Discussion section. The characteristics defining the two different populations sampled may explain some of the significant jumps in improved health between the older Irish studies and the current studies in spite of the fact that the current OHA subgroup is representative of the TILDA study overall (Table 1). As a result the conclusions from this study may not be generalizable to the entire Irish population, especially those not living in the urban capital of the country.

**Comments on Conclusions:**

 Conclusions should be based on the work and findings presented in this manuscript. Yet the authors say, “Currently, there is little service provision for dentate older adults, especially those who become frail or dependent, suggesting the need for a qualitative change in the oral care for older people in Ireland.” I am not sure why this statement is made in the Conclusion of the paper as this topic was not a part of the study and cannot be concluded from the data collected.

**Closing Thoughts:**

 In the beginning of the article, the authors discuss the importance of this data to better inform policy makers in the development of a new national health care policy; however, little data was collected on the truly elderly population. This is a missed opportunity as limited health care dollars require that the at-risk population be studied to enable targeting of limited funds to cohorts at greatest need. I encourage future OHA studies, whether as part of TILDA or not, to do a better job of oversampling, especially the middle and oldest groups of elderly (75+ and 85+ years of age) and those who are living in a multitude of living arrangements and geographic settings, so that the most accurate picture of oral disease in Irish elderly is captured.

Is the work clearly and accurately presented and does it cite the current literature?

Yes

If applicable, is the statistical analysis and its interpretation appropriate?

Partly

Are all the source data underlying the results available to ensure full reproducibility?

Yes

Is the study design appropriate and is the work technically sound?

Partly

Are the conclusions drawn adequately supported by the results?

Partly

Are sufficient details of methods and analysis provided to allow replication by others?

Yes

Reviewer Expertise:

NA

I confirm that I have read this submission and believe that I have an appropriate level of expertise to confirm that it is of an acceptable scientific standard.

# Peer review: Reviewer response for version 1

**DOI:** 10.21956/hrbopenres.13960.r26444

**Published:** 2019-01-09

**Authors:** W. Murray Thomson

**Affiliations:** 1 Department of Oral Sciences, Sir John Walsh Research Institute, Faculty of Dentistry, University of Otago, Dunedin, New Zealand

**General comments**

 Paper reports the findings of a simple descriptive analysis of oral epi data from participants in a cohort study of adults aged 50+. The title and abstract refer to “oral health status”, but what is actually being described here is tooth retention, dental caries experience and a snapshot of periodontal status, rather than “oral health” per se. The presentation of findings is not as systematic and easy to follow as it should be, because the authors give the impression of not being sure whether this paper will be a simple overview of the oral characteristics of the sample or a comparison of changes over time – and the Results section reflects that, with data in the later stages being brought in from earlier Irish work and from overseas studies. I suggest that they confine the earlier Irish work and overseas data to the Discussion. A purely descriptive analysis like this one should be carefully and systematically presented, with a clear boundary between the study data and their comparison with earlier findings.

 The authors insist on using the awful “compared with”; I suggest that they desist. Thus, “Of those aged 65 years and older, 15.6% were dentate compared with 40.9% in 2000-02” should be written as “Of those aged 65 years and older, 15.6% were dentate, while it was 40.9% in 2000-02” (or somesuch).

 There are too many acronyms – do we really need OHA and MIP?

 There are far too many small Tables – the authors should concentrate on having larger, more efficient Tables where the sociodemographic (and other) associations are presented to the reader.

**Section comments**

*Title*

 The title is unnecessarily long–a shorter and more appropriate one is indicated.

*Abstract*

 This will need to be rewritten once the paper has been revised, but the authors should consider the following: do not leave the reader to calculate %; be consistent with the number of decimal places for the % that you do present (these should be to 1 decimal place); do not use a hackneyed phrase such as “the results suggest”; and the conclusion should be a valid conclusion (what’s the take-home message from the study?).

*Introduction*

 This is rather long and meandering. There are contentious assertions in paragraph 2 which should be omitted or tempered. The first clause of sentence 2 is vague – what do the AU mean here? Similarly, sentence 3 is unnecessary and can be contested – the great bulk of the evidence for the so-called “bidirectional relationship” is weak, fails to account for confounders, and is mostly the outcome of wishful thinking in interpreting cross-sectional data. Oral health is important enough in its own right without our having to try to justify investigating it using spurious associations with general health conditions.

 In para 3, sentence 3 has structural problems and should be rewritten.

 The last para of the Intro is actually Discussion and does not belong in the Intro.

*Methods*

 There is insufficient information on the sampling procedures and what the sampling frame was. We are not told in the right place in Methods whether it is a simple random sample or a complex sample. Is it a national sample, or just people in and around Dublin? Also, these were people in Wave 3 – does this mean that they were also assessed earlier in Waves 1 and 2? If so, we need more information on that, and particularly on participation rates and loss to follow-up in earlier assessments.

 Hyphenate “cross infection”.

 The assessment of tooth contacts in the dentition does not actually mention the maxillary teeth – I presume that they are referring to contacts between the maxillary and mandibular teeth. And the term “functionality of the dentition” is unusual.

 The RCI is not a measure of root surface caries prevalence, despite Katz having used that term in his original paper. The prevalence of a condition is the proportion of the population who have the condition – that should be reported here, alongside the RCI and the mean number of root DFT. The RCI is a sort of “attack rate” indicator.

 Use the term “denture wearing”, not “denture wear” – that’s something else entirely…

*Results*

 As a general principle, have one paragraph of Results text per Table and do not repeat Table data in the accompanying text (instead, summarise). Do not discuss your findings in the Results section (e.g. Results para 3, sentence 2 is Discussion).

 Re: the data in Table 1 – describe the Table as reporting the sample characteristics, etc – the actual statistical testing is secondary. While on the latter, the authors do themselves a disservice, I think – while a lot of those P values are <0.05, this is due to the large N rather than any meaningful differences in proportions. For example, 27.1% and 24.1% living in Dublin – each of those I would summarise as approximately 1 in 4. Doing the analyses by each category of age group (or education level, etc) means a lot of tests – why not one cross-tabulation for each domain rather than 3?

 Results para 4, last sentence – you cannot say that the gender difference “decreased with increasing age” – it was less marked in those who were older; you did not see it decrease… This is actually a surprising finding, by the way – in most epi studies of older people, you will see higher proportions of females in the older age groups. Why is yours different?

 Table 3 should also present the edentulous and dentate proportions for each age group (genders combined). Table 4 is not really a stand-alone Table – consider presenting that information in Table 3 as well – you are going to have to restructure it anyway. Again, you cannot say (as you do at the end of page 4) that the proportion of edentate adults increased with age – again, it was higher in those who were older.

 Table 5 should refer to denture wearing (see above!). Again, why repeat the table data in the text? We can see the Table, so point out the highlights. Again, there is the problem phrase “increased with age” (see above)…

 Figure 1 – is this necessary? (ditto Figures 2, 3 and 4 – especially Figure 4…). Whereas Tables are a very efficient way of presenting a lot of information, Figures are not, and that information can be summarised and presented efficiently in the Results text – or as a Table, with mean numbers and SDs presented by sociodemographic characteristics – that would certainly be more informative. The last sentence of the “number of teeth” section is actually Discussion – and two data points do not make a “trend”.

 To the periodontal data – the CPI is a bad index to use anyway, and even worse when used in a longitudinal study. It’s too late now to point out that attachment loss data should have been collected. Again, the presentation of the data is not as informative as it might have been.

*Discussion*

 See my earlier comments (page 1).

 The last para of the Discussion misuses the term “extrapolated” – that should be “generalised”.

Is the work clearly and accurately presented and does it cite the current literature?

Partly

If applicable, is the statistical analysis and its interpretation appropriate?

Partly

Are all the source data underlying the results available to ensure full reproducibility?

No source data required

Is the study design appropriate and is the work technically sound?

Yes

Are the conclusions drawn adequately supported by the results?

Partly

Are sufficient details of methods and analysis provided to allow replication by others?

Partly

Reviewer Expertise:

Dental epidemiology, gerodontology, health services research

I confirm that I have read this submission and believe that I have an appropriate level of expertise to confirm that it is of an acceptable scientific standard, however I have significant reservations, as outlined above.

# Response

**Published:** 2021-08-18

**Authors:** Brian O'Connell

**Affiliations:** Dublin Dental University Hospital, Ireland

**General comments**

 Paper reports the findings of a simple descriptive analysis of oral epi data from participants in a cohort study of adults aged 50+. The title and abstract refer to “oral health status”, but what is actually being described here is tooth retention, dental caries experience and a snapshot of periodontal status, rather than “oral health” per se. The presentation of findings is not as systematic and easy to follow as it should be, because the authors give the impression of not being sure whether this paper will be a simple overview of the oral characteristics of the sample or a comparison of changes over time – and the Results section reflects that, with data in the later stages being brought in from earlier Irish work and from overseas studies. I suggest that they confine the earlier Irish work and overseas data to the Discussion. A purely descriptive analysis like this one should be carefully and systematically presented, with a clear boundary between the study data and their comparison with earlier findings.

*Thank you for your helpful review and comments. We have done our best to reflect these suggestions in the revised version of the paper. Throughout the paper and in the title ‘oral health’ has been changed to ‘dental health’ and we have limited comparisons with previous and international data to the Discussion section.*

 The authors insist on using the awful “compared with”; I suggest that they desist. Thus, “Of those aged 65 years and older, 15.6% were dentate compared with 40.9% in 2000-02” should be written as “Of those aged 65 years and older, 15.6% were dentate, while it was 40.9% in 2000-02” (or somesuch).

*This has been amended throughout the document*

 There are too many acronyms – do we really need OHA and MIP?

*We have removed OHA throughout the document and reduced the use of other acronyms as much as possible* 

 There are far too many small Tables – the authors should concentrate on having larger, more efficient Tables where the sociodemographic (and other) associations are presented to the reader.

*We have considered your suggestion about reporting the relationship between dental health and socio-economic status. This paper is already very long so we have decided to publish a separate paper on this topic by using socioeconomic variables from TILDA dataset. We have removed Table 4.*

**Section comments**

*Title*

 The title is unnecessarily long–a shorter and more appropriate one is indicated.

*We understand the reviewer’s comments but the publisher requires a full description of the study type in the title according to the STROBE guidelines*

*Abstract*

 This will need to be rewritten once the paper has been revised, but the authors should consider the following: do not leave the reader to calculate %; be consistent with the number of decimal places for the % that you do present (these should be to 1 decimal place);

*The percentages reported have been checked and all are now at one decimal place* *.*

 do not use a hackneyed phrase such as “the results suggest”; and the conclusion should be a valid conclusion (what’s the take-home message from the study?).

*Changes has been made in the conclusions section to focus on the relevance of the findings of this cross-sectional study. We have removed the phrase ‘the results suggest’*

*Introduction*

 This is rather long and meandering. There are contentious assertions in paragraph 2 which should be omitted or tempered. The first clause of sentence 2 is vague – what do the AU mean here? Similarly, sentence 3 is unnecessary and can be contested – the great bulk of the evidence for the so-called “bidirectional relationship” is weak, fails to account for confounders, and is mostly the outcome of wishful thinking in interpreting cross-sectional data. Oral health is important enough in its own right without our having to try to justify investigating it using spurious associations with general health conditions.

*These suggestions have been included in the revisions.  The focus in the introduction is on dental health indicators and any association between dental health and systemic health.*

 In para 3, sentence 3 has structural problems and should be rewritten.

*This paragraph has been rewritten and is more focused.*

 The last para of the Intro is actually Discussion and does not belong in the Intro.

*The last paragraph has been removed*

*Methods*

 There is insufficient information on the sampling procedures and what the sampling frame was. We are not told in the right place in Methods whether it is a simple random sample or a complex sample. Is it a national sample, or just people in and around Dublin? Also, these were people in Wave 3 –

*We have given more information in the text to clarify these points.*

 does this mean that they were also assessed earlier in Waves 1 and 2? If so, we need more information on that, and particularly on participation rates and loss to follow-up in earlier assessments.

*The dental health assessment was introduced in TILDA Wave 3 so we only have cross-sectional data. However, we are aiming to repeat dental assessment in the next TILDA wave to have longitudinal data.*

 Hyphenate “cross infection”.

*This has been corrected.*

 The assessment of tooth contacts in the dentition does not actually mention the maxillary teeth – I presume that they are referring to contacts between the maxillary and mandibular teeth. And the term “functionality of the dentition” is unusual.

*That is correct—tooth contacts (in Maximum Intercuspal Position)  means contact between opposing pairs of teeth--this has been clarified in the text. The more usual term ‘functional dentition’ has been used to express the concept of a dentition that is adequate for normal function.*  

 The RCI is not a measure of root surface caries prevalence, despite Katz having used that term in his original paper. The prevalence of a condition is the proportion of the population who have the condition – that should be reported here, alongside the RCI and the mean number of root DFT. The RCI is a sort of “attack rate” indicator.

*Yes, we agree.  It represents the proportion of exposed root surfaces with root caries and it was used to allow for comparisons to be made with previous Irish national surveys.  The tables also report the mean number of teeth with decayed and filled roots. The text has been changed in the Methods section to clarify the meaning of the RCI.*

 Use the term “denture wearing”, not “denture wear” – that’s something else entirely…

*The term has been changed to ‘denture wearing’.*

*Results*

 As a general principle, have one paragraph of Results text per Table and do not repeat Table data in the accompanying text (instead, summarise). Do not discuss your findings in the Results section (e.g. Results para 3, sentence 2 is Discussion).

*The Results section has been revised as recommended.*

 Re: the data in Table 1 – describe the Table as reporting the sample characteristics, etc

*The title of the table has been changed as suggested.*

 the actual statistical testing is secondary. While on the latter, the authors do themselves a disservice, I think – while a lot of those P values are <0.05, this is due to the large N rather than any meaningful differences in proportions. For example, 27.1% and 24.1% living in Dublin – each of those I would summarise as approximately 1 in 4. Doing the analyses by each category of age group (or education level, etc) means a lot of tests – why not one cross-tabulation for each domain rather than 3?

*In order to compare the characteristics of the two different-sized samples--dental health and total TILDA -- the two-sample proportion test was chosen (following the advice of two independent experienced biostatisticians).*

*For the cross tabulation we measured the relationship between different categories of two variables. Although the proportions were not much different between the full TILDA and the dental samples, the Z test showed similarities between the two samples for 8 out of 23 characteristics (Table 1).*

 Results para 4, last sentence – you cannot say that the gender difference “decreased with increasing age” – it was less marked in those who were older; you did not see it decrease… *We understand this wording was misleading so all wording in results has been changed from decreased with age to the reporting of comparison between two age groups.*

 This is actually a surprising finding, by the way – in most epi studies of older people, you will see higher proportions of females in the older age groups. Why is yours different?

*This may be because the dental sample is a convenience sample, unlike the TILDA cohort, which is nationally representative.*

 Table 3 should also present the edentulous and dentate proportions for each age group (genders combined).

*Given the finding in previous national surveys that the prevalence of edentulism was much higher in females (Table 12), it was considered notable to demonstrate the change in this trend over time and among the different age cohorts in the present study*

  Table 4 is not really a stand-alone Table – consider presenting that information in Table 3 as well –

*Table 4 has been deleted and the main results given in the text.*

 you are going to have to restructure it anyway. Again, you cannot say (as you do at the end of page 4) that the proportion of edentate adults increased with age – again, it was higher in those who were older.

*This wording has been changed throughout the document.*

 Table 5 should refer to denture wearing (see above!). Again, why repeat the table data in the text? We can see the Table, so point out the highlights. Again, there is the problem phrase “increased with age” (see above)…

*As reported above, it has been changed*

 Figure 1 – is this necessary? (ditto Figures 2, 3 and 4 – especially Figure 4…). Whereas Tables are a very efficient way of presenting a lot of information, Figures are not, and that information can be summarised and presented efficiently in the Results text – or as a Table, with mean numbers and SDs presented by sociodemographic characteristics – that would certainly be more informative.

*As the data was not categorical or normally distributed, we believe the figures give added information regarding the data distribution* *. This was limited to four figures, but can be reduced further if required.*

*We agree with the suggestion, also mentioned previously, to publish a separate paper on dental health according to sociodemographic characteristics.*

 The last sentence of the “number of teeth” section is actually Discussion

*This has been removed* – and two data points do not make a “trend”.

*We understand the wording was misleading so it has been changed.*

 To the periodontal data – the CPI is a bad index to use anyway, and even worse when used in a longitudinal study. It’s too late now to point out that attachment loss data should have been collected. Again, the presentation of the data is not as informative as it might have been.

*Yes, we understand CPI is not an ideal index for longitudinal studies but the dental assessment was allocated only 10 minutes in the study, which was not enough time to measure attachment loss. Also, there was only time to measure index teeth. We have requested a more comprehensive periodontal measurements in the next wave of TILDA, but as the whole health assessment takes three hours or more, this remains to be decided.  We understand the onerous nature of a lengthy assessment for respondents, some of whom are frail and do not even understand why a dental assessment is part of the health assessment! But that is another matter*.

*Discussion*

 See my earlier comments (page 1). 

*The Discussion has been revised according to these suggestions*

 The last para of the Discussion misuses the term “extrapolated” – that should be “generalised”.

*The wording has been changed*

*Thank you again for your in depth and valuable input. We hope the changes described above have improved the paper and addressed your significant reservations.*
